# Acid ceramidase regulates innate immune memory

**DOI:** 10.1016/j.celrep.2023.113458

**Published:** 2023-11-22

**Authors:** Nils Rother, Cansu Yanginlar, Geoffrey Prévot, Inge Jonkman, Maaike Jacobs, Mandy M.T. van Leent, Julia van Heck, Vasiliki Matzaraki, Anthony Azzun, Judit Morla-Folch, Anna Ranzenigo, William Wang, Roy van der Meel, Zahi A. Fayad, Niels P. Riksen, Luuk B. Hilbrands, Rik G.H. Lindeboom, Joost H.A. Martens, Michiel Vermeulen, Leo A.B. Joosten, Mihai G. Netea, Willem J.M. Mulder, Johan van der Vlag, Abraham J.P. Teunissen, Raphaël Duivenvoorden

**Affiliations:** 1 Department of Nephrology, Research Institute for Medical Innovation, Radboud University Medical Center, Nijmegen, the Netherlands; 2 Biomolecular Engineering and Imaging Institute, Icahn School of Medicine at Mount Sinai, New York, NY, USA; 3 Cardiovascular Research Institute, Icahn School of Medicine at Mount Sinai, New York, NY, USA; 4 Department of Medical Biochemistry, Amsterdam University Medical Centers, Amsterdam, the Netherlands; 5 Department of Internal Medicine and Radboud Center for Infectious Diseases, Radboud University Medical Center, Nijmegen, the Netherlands; 6 Laboratory of Chemical Biology, Department of Biomedical Engineering and Institute for Complex Molecular Systems, Eindhoven University of Technology, Eindhoven, the Netherlands; 7 Department of Molecular Biology, Faculty of Science, Oncode Institute, Radboud University Nijmegen, Nijmegen, the Netherlands; 8 Department of Medical Genetics, University of Medicine and Pharmacy, Iuliu Hat‚ieganu, Cluj-Napoca, Romania; 9 Department of Immunology and Metabolism, Life and Medical Sciences Institute, University of Bonn, Bonn, Germany; 10 Icahn Genomics Institute, Icahn School of Medicine at Mount Sinai, New York, NY, USA; 11 These authors contributed equally; 12 Lead contact

## Abstract

Innate immune memory, also called “trained immunity,” is a functional state of myeloid cells enabling enhanced immune responses. This phenomenon is important for host defense, but also plays a role in various immune-mediated conditions. We show that exogenously administered sphingolipids and inhibition of sphingolipid metabolizing enzymes modulate trained immunity. In particular, we reveal that acid ceramidase, an enzyme that converts ceramide to sphingosine, is a potent regulator of trained immunity. We show that acid ceramidase regulates the transcription of histone-modifying enzymes, resulting in profound changes in histone 3 lysine 27 acetylation and histone 3 lysine 4 trimethylation. We confirm our findings by identifying single-nucleotide polymorphisms in the region of *ASAH1*, the gene encoding acid ceramidase, that are associated with the trained immunity cytokine response. Our findings reveal an immunomodulatory effect of sphingolipids and identify acid ceramidase as a relevant therapeutic target to modulate trained immunity responses in innate immune-driven disorders.

## In brief

The sphingolipid metabolism is known to regulate cellular functions, including immune cell regulation. Rother et al. show that inhibition of acid ceramidase, a sphingolipid-converting enzyme, prevents the induction of trained immunity. This identifies an immunomodulatory function of sphingolipid metabolism in regulating the immunologic memory of innate immune cells.

## Graphical Abstract

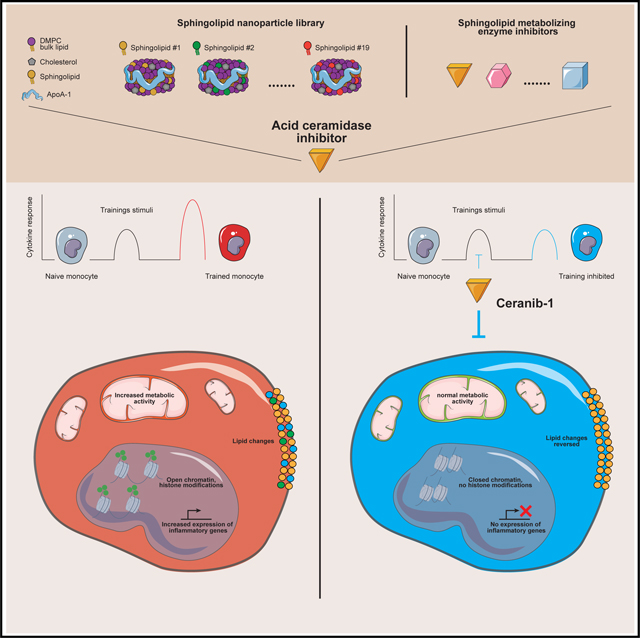

## INTRODUCTION

Until recently, immunologic memory was considered an exclusive feature of the adaptive immune system. However, this view has been challenged by the discovery that innate immune cells also adapt and harness a form of immunologic memory. After exposure to certain stimuli, monocytes and macrophages can develop a quicker and stronger inflammatory response to subsequent unrelated stimuli.^1^ This innate immune memory, also termed trained immunity, relies on epigenetic reprogramming, specifically changes in histone modifications (e.g., H3K4me3, H3K27ac), that facilitates the transcription of inflammatory genes.^2^ The reverse can also occur, where a stimulus induces epigenetic silencing of inflammatory gene transcription and innate immune cells acquire a tolerized state.^3,4^ Trained immunity is important for host defense, but also has maladaptive features in immune-mediated conditions such as atherosclerosis, cancer, autoinflammatory disorders, and organ transplantation.^5–9^

The epigenetic modifications underlying trained immunity are closely linked to metabolic changes, including the induction of aerobic glycolysis, glutaminolysis, and cholesterol metabolism.^8,10,11^ Intermediates of these metabolic pathways serve as substrates for epigenetic enzymes or as regulators of these enzymes. The mechanistic target of rapamycin (mTOR) orchestrates these metabolic pathways, making it a key regulator of trained immunity.^10^ This complex interaction between metabolism and the epigenetic machinery enables innate immune cells to coordinate their energy expenditure and inflammatory activity. Thorough insight into the metabolic regulation of the epigenome is critical for discovering therapeutic targets to modulate trained immunity.

Previous studies of metabolic and epigenetic reprogramming in trained immunity hinted at lipid metabolism as a regulatory metabolic circuit.^3,11^ Here, we investigate the role of a specific class of lipids, namely sphingolipids, in trained immunity. Sphingolipids are an extended family of bioactive lipids that are universally present in eukaryotes and possess immunomodulatory properties.^12^ By altering sphingolipid levels through either exogenously administering sphingolipid-loaded nanobiologics or by inhibiting key enzymes in sphingolipid metabolism we show that innate immune cells can be modulated toward a trained or tolerized state. By combining lipidomic, transcriptomic, and epigenetic studies we identified acid ceramidase, the enzyme catalyzing the conversion of ceramide to sphingosine, as an essential regulator of trained immunity. We validated this finding with a functional trained immunity quantitative trait loci (FTI-QTL) analysis in the 300BCG cohort of the Human Functional Genomics Project.^13^

## RESULTS

### Sphingolipids modulate trained immunity

**Figure 1.**
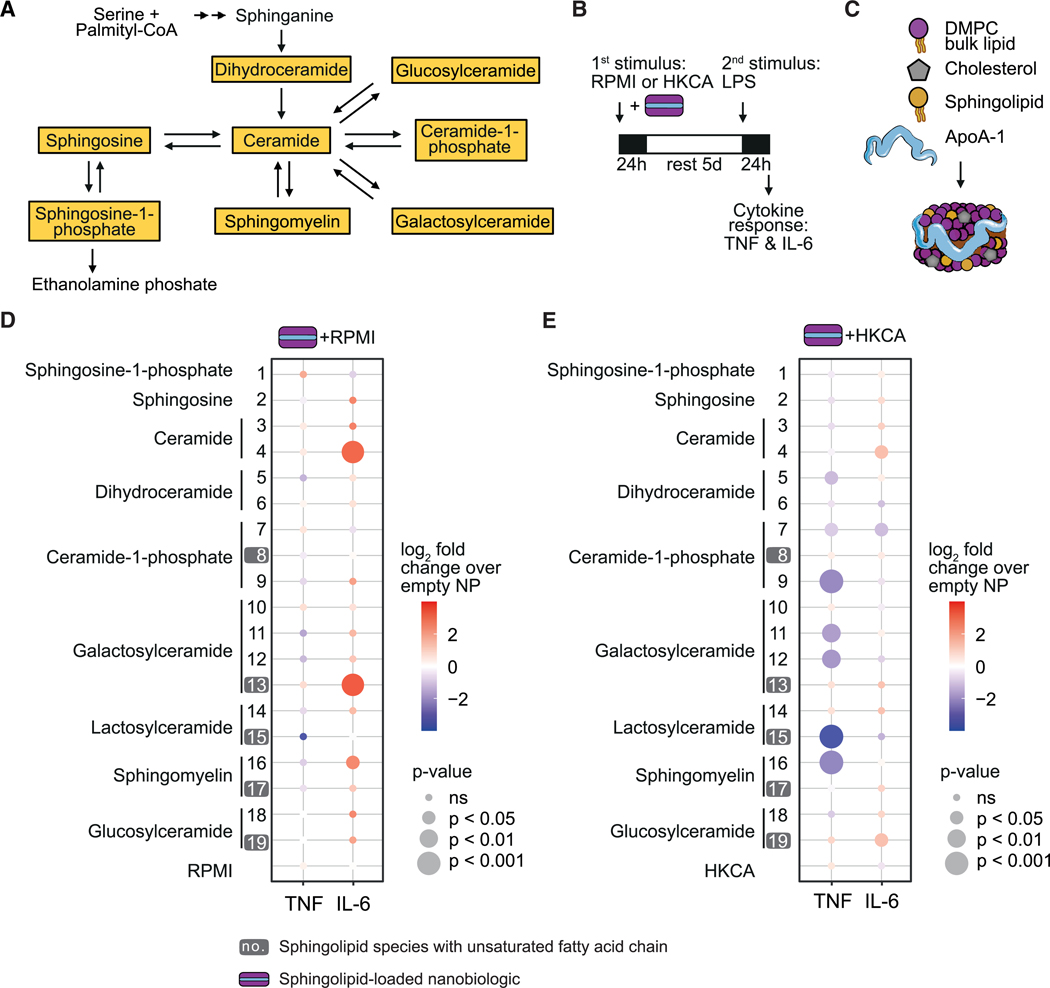
Sphingolipid-loaded nanobiologics modulate trained immunity (A) Schematic depiction of the predominant sphingolipid classes and their metabolic interconversion. Yellow boxes indicate sphingolipid classes that were incorporated in the nanobiologics. (B) Schematic depiction of the *in vitro* trained immunity assay performed using the sphingolipid-loaded nanobiologics. (C) Schematic depiction of sphingolipid-loaded nanobiologic composition. (D and E) PBMCs were stimulated for 24 h with sphingolipid-loaded nanobiologics (50 μM) alone (D) or in combination with HKCA (E). After a 5-day resting period, cells were restimulated with LPS for 24 h and cytokine production measured in the supernatant by ELISA (n = 6 donors). Data are expressed as log2 fold change compared with empty NPs in (D) or compared with empty NPs + HKCA in (E). p Values were calculated using a one-way ANOVA with Dunnett’s post-test of the sphingolipid nanoparticle versus RPMI in (D) and HKCA in (E). For bar graphs with absolute cytokine concentrations of data in (D and E) see Figures S1B–S1E. DMPC, 1,2-dimyristoyl-*sn*-glycero-3-phosphocholine. P values calculated for the sphingolipid nanoparticles versus RPMI or HKCA can be found in Table S2. See also Tables S1 and S2 and Figure S1.

Sphingolipids are evolutionary ancient structures comprising a broad variety of interconvertible subspecies.^12^ *De novo* synthesis of sphingolipids leads to the formation of ceramide, which serves as a precursor to various types of other sphingolipids (Figure 1A). These lipid molecules regulate basic cellular processes, including growth, adhesion, migration, apoptosis, and senescence.^14^ In addition, sphingolipids play a critical role in shaping immune responses.^14,15^

We set out to investigate the role of sphingolipids in trained immunity. For this purpose, we adapted an *in vitro* protocol in which human peripheral blood mononuclear cells (PBMCs) are stimulated for 24 h, followed by a 5-day resting period and a subsequent 24-h restimulation phase. This is a well-established *in vitro* assay to investigate trained immunity, described in great detail by Bekkering and co-workers, and has been applied extensively in previous studies.^1–3,16–18^ We used heat-killed *Candida albicans* (HKCA) to induce trained immunity and Toll-like receptor 4 (TLR4) agonist lipopolysaccharide (LPS) as a secondary stimulus.^18^ The induction of trained immunity is assessed by measuring interleukin-6 (IL-6) and tumor necrosis factor (TNF) production in the supernatant (Figure 1B).^16^

First, we investigated the effect of exogenously administered sphingolipids on trained immunity in human primary monocytes. To facilitate efficient delivery of sphingolipids to monocytes, we used a nanobiologic delivery platform that targets myeloid cells with high efficiency.^19–21^ Nanobiologics are nanocarriers consisting of exclusively natural building blocks, namely lipids (phospholipids, triglycerides, cholesterol) and apolipoprotein A (apoA-1), the main protein constituent of high-density lipoprotein. Nanobiologics’ intrinsic propensity for myeloid cell uptake allows an efficient delivery of lipophilic compounds to the innate immune system. We designed a nanotherapeutic library containing nanobiologics loaded with 19 different sphingolipids (Figure 1C; Table S1). All nanobiologic formulations were non-toxic to PBMCs at the concentrations used (Figure S1A). PBMCs were either stimulated with sphingolipid-loaded nanobiologics alone (Figures 1D, S1B, and S1C) or in combination with HKCA (Figures 1E, S1D, and S1E) for 24 h and restimulated with LPS 5 days later. We observed that three sphingolipid-loaded nanobiologics (containing d18:1/16:0 ceramide [no. 4], d18:1/24:1 galactosyl(β) ceramide [no. 13], or 24:0 sphingomyelin [no. 16]) enhanced IL-6 production upon restimulation, indicating that these formulations induce trained immunity (Figures 1D and S1C). Two sphingolipid-loaded nanobiologics (containing d18:1/16:0 ceramide [no. 4] and d18:1/18:1 glucosyl(β) ceramide [no. 19]) augmented the HKCA-induced trained immune response for IL-6 (Figures 1E and S1E).). Seven sphingolipid-loaded nanobiologics (containing d18:0/16:0 dihydroceramide [no. 5], d18:1/16:0 ceramide-1-phosphate [no. 7], d18:1/12:0 ceramide-1-phosphate [no. 9], d18:1/16:0 and d18:1/24:0 galactosyl(β) ceramide [nos. 11 and 12], d18:1/24:1 lactosyl(β) ceramide [no. 15], and 24:0 sphingomyelin [no. 16]) inhibited the HKCA-induced trained immune response as indicated by a reduction in TNF production (Figures 1E and S1D).

These data demonstrate that even a short exposure to some of the exogenously administered sphingolipids can induce marked changes in the function of innate immune cells, indicative of a trained immunity response. Interestingly, this process depends not only on the type of sphingolipid headgroup, but also the saturation status of its fatty acid residue. Species with a saturated fatty acid chain (nos. 12 and 14) had an opposite effect on trained immunity compared with their *cis*-monounsaturated counterparts (nos. 13 and 15), when added together with HKCA (Figure 1E). In addition, inhibiting trained immunity using sphingolipid-loaded nanobiologics had stronger effects on TNF production than on IL-6 secretion. In contrast, induction of trained immunity was most clearly observed in the IL-6 response.

### Inhibiting sphingolipid metabolizing enzymes affects trained immunity

**Figure 2.**
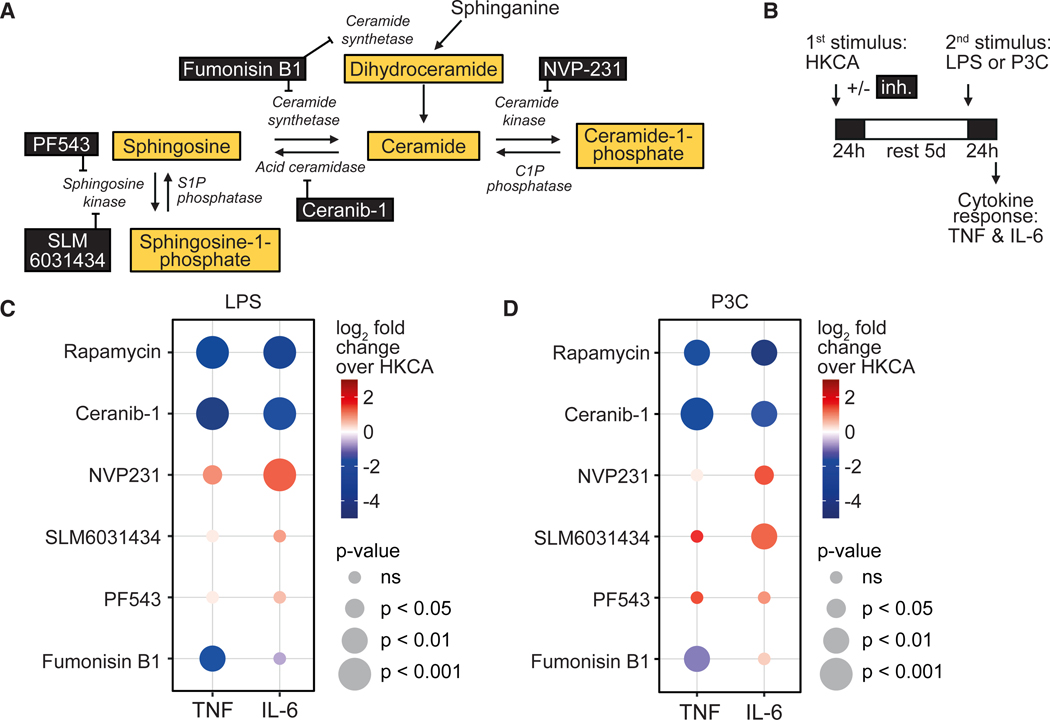
Modulating sphingolipid metabolism affects trained immunity (A) Schematic representation of sphingolipid metabolism, including key enzymes (italic) and the enzyme inhibitors employed in this study (black boxes). (B) Schematic depiction of the *in vitro* trained immunity assay performed using the sphingolipid-relevant enzyme inhibitors. (C and D) PBMCs were stimulated for 24 h with HKCA alone, with HKCA together with specified inhibitors (rapamycin 0.1 μM, ceranib-1 10 μM, NVP231 1 μM, SLM6031434 1 μM, PF534 1 μM, fumonisin B1 500 μM) or RPMI as control. After a 5-day resting period, cells were restimulated for 24 h with LPS (C) or Pam3CSK (P3C) (D) and cytokine production measured in the supernatant by ELISA (n = 4 donors for fumonisin B1 and NVP231, n = 6 donors for ceranib-1, rapamycin, PF543, and SLM6031434). Data are represented as log2 fold change compared with HKCA-trained cells. For bar graphs with absolute cytokine concentrations of data in (C and D) see Figures S2B and S2C. See also Figures S2–S8.

Next, we assessed whether metabolism of sphingolipids affects trained immunity. For this purpose, we selected five small-molecule inhibitors of enzymes critical in sphingolipid metabolism. PF543 inhibits sphingosine kinase 1 (*SPHK1*), SLM6031434 inhibits sphingosine kinase 2 (*SPHK2*), ceranib-1 inhibits acid ceramidase (*ASAH1*), fumonisin B1 inhibits ceramide synthetase (*CERS*), and NVP231 inhibits ceramide kinase (*CERK*) (Figure 2A). Dose finding experiments were performed to determine the maximum non-toxic concentration of each inhibitor, defined as <15% lactate dehydrogenase (LDH) release after 24 h (Figures S2A–S2C). The mTOR inhibitor rapamycin was used as a positive control that is known to inhibit trained immunity.^10^ PBMCs were stimulated with HKCA with or without the addition of a small-molecule inhibitor for 24 h and restimulated 5 days later with LPS or Pam3CSK (P3C) for TLR4 and TLR2 stimulation, respectively (Figure 2B). The inhibitors had diverse effects on trained immunity (Figures 2C, 2D, S3A, S3B, and S4). Ceranib-1 inhibited trained immunity with a potency similar to rapamycin for both IL-6 and TNF production. To evaluate whether this effect is mediated by changes in sphingosine or sphingosine-1-phosphate (S1P), we tested the effects of SLM6031434 and PF543 on trained immunity. SLM6031434 increased trained immunity mainly for IL-6 production. PF543 showed no effect (Figures 2C, 2D, S3A, and S3B). Adding sphingosine or S1P-containing nanobiologics could not rescue the training in ceranib-1-treated cells (Figure S5). Together, this indicates that the effects of ceranib-1 are likely not mediated by lowering sphingosine or S1P. Fumonisin B1 inhibited trained immunity only for the TNF response. In contrast, NVP231 augmented trained immunity primarily for the IL-6 production. The effects observed were mostly similar for secondary stimulations with LPS or P3C, indicating that modulating sphingolipid metabolism changes the trained immunity status of monocytes in such a way that both TLR2 and 4 stimulations are affected.

The inhibition of acid ceramidase by ceranib-1 showed the strongest and most consistent effect on trained immunity. The effects of ceranib-1 on trained immunity were not accompanied by effects on monocyte differentiation, expression of activation markers or the induction of cell death (Figures 6 and S7). Whereas training with HKCA led to a slight increase in monocyte cell numbers that were mainly of the intermediate phenotype (CD14^+^/CD16^+^, Figure S6B) and an increase in HLA-DR expression (Figure S6C), ceranib-1 did not reverse this. In addition, ceranib-1 did not induce apoptosis (AnnexinV^+^), necrosis (AnnexinV^+^/PI^+^) after 24 h or after the resting period (Figures S7A–S7C). LDH was also not increased after the resting period (Figure S7D). Reseeding equal amounts of monocytes before restimulation resulted in equally potent inhibition of trained immunity by ceranib-1 (Figure S7E). The potency of inhibiting acid ceramidase is supported by the use of two other acid ceramidase inhibitors, similarly showing inhibition of trained immunity cytokine responses at non-toxic levels (Figure S8). These data show that inhibiting the key enzyme in sphingolipid metabolism strongly influences the induction of trained immunity. Acid ceramidase inhibition by ceranib-1 showed the most consistent effect. This incentivized us to further unravel the mechanism through which acid ceramidase regulates trained immunity.

### Inhibiting acid ceramidase reprograms immunometabolism

**Figure 3.**
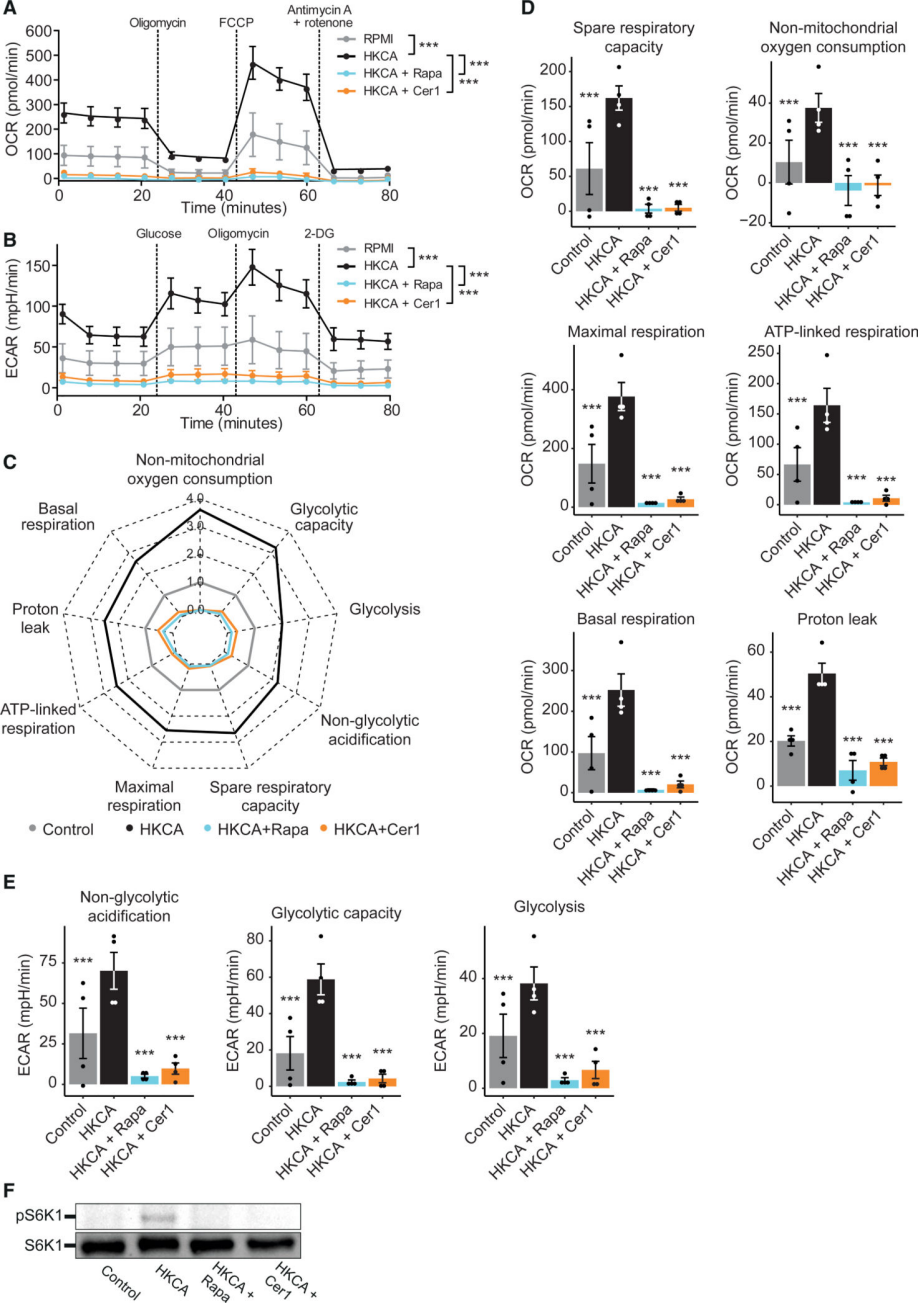
Inhibition of acid ceramidase blocks monocyte metabolism (A–E) PBMCs were stimulated for 24 h with HKCA alone, HKCA with rapamycin (Rapa) or ceranib-1 (Cer1), or RPMI as control. After 5 days of rest, cellular metabolic activity was assessed by Seahorse analysis (n = 4 donors). Metabolic parameters as displayed in (C–E) were calculated from the oxygen consumption rate (OCR) (A) and extracellular acidification rate (ECAR) (B) as detailed in the STAR Methods. Data are represented as mean ± SEM in (A, B, D, and E) and as mean fold change compared with RPMI in (C). (F) PBMCs were stimulated for 24 h with HKCA alone, with HKCA and rapamycin or ceranib-1, or RPMI as control. The phosphorylation of the mTOR target S6K1, was analyzed using western blot analysis. Representative blot of two replicates. ***p < 0.001; p values were calculated using a one-way ANOVA with Dunnett’s post-test compared with HKCA.

The epigenetic changes underlying trained immunity are mediated by metabolic reprogramming of innate immune cells.^8,10^ Therefore, we proceeded by investigating acid ceramidase’s role in immunometabolism. For this purpose, we assessed the effect of ceranib-1 on cell metabolism *in vitro* in HKCA-trained PBMCs by extracellular flux analysis using Seahorse technology. We used the mTOR inhibitor rapamycin again as a well-established inhibitor of trained immunity. Changes in oxygen consumption rate in response to oligomycin (OM), carbonyl cyanide 4-(trifluoromethoxy)phenylhydrazone, and rotenone + antimycin A injection were used to calculate oxidative phosphorylation parameters (Figures 3A and 3C). Variations in the extracellular acidification rate in response to glucose and OM injection were used to calculate glycolysis parameters (Figures 3B and 3C). We found that acid ceramidase inhibition markedly suppresses both oxidative phosphorylation (Figure 3D) and glycolysis (Figure 3E) compared with HKCA-trained cells.

The metabolic effects of acid ceramidase inhibition were similar to those of mTOR inhibition, which led us to assess if acid ceramidase inhibition affects mTOR signaling. Therefore, we stimulated PBMCs for 24 h with HKCA or RPMI as control, and visualized the phosphorylation of the mTOR target S6K1 by western blot analysis. HKCA-trained cells showed S6K1 phosphorylation, while this was not visible in untrained cells (RPMI) (Figure 3F). Ceranib-1 suppressed S6K1 phosphorylation similar to rapamycin, indicating that acid ceramidase inhibition downregulates mTOR activity.

Together, these data reveal a potent effect of acid ceramidase inhibition on immunometabolism, which may partly be mediated by inhibition of mTOR signaling.

### Inhibiting acid ceramidase modulates the monocyte lipidome

**Figure 4.**
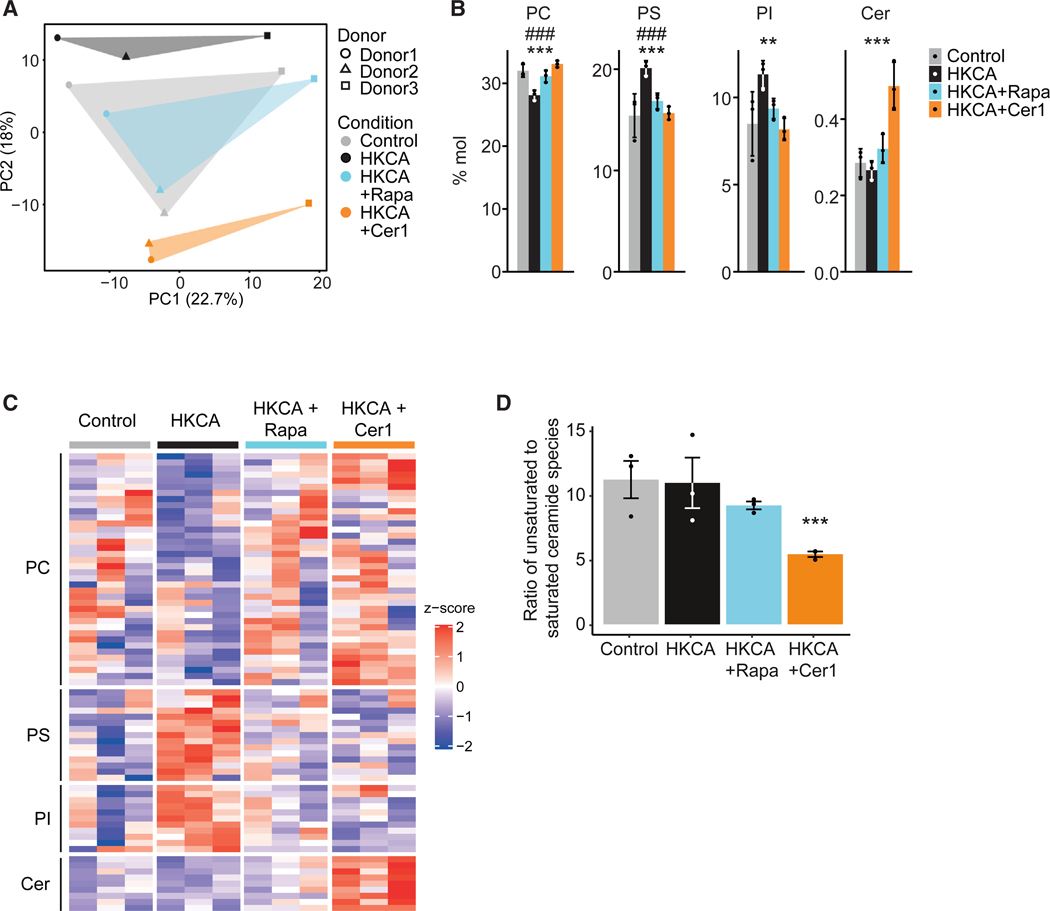
Acid ceramidase inhibition alters the monocyte’s lipidome (A–D) PBMCs were stimulated for 24 h with HKCA alone, with HKCA and rapamycin (Rapa) or ceranib-1 (Cer1), or RPMI as control. Subsequently, monocytes were purified and their lipid content analyzed by mass spectrometry-based shotgun lipidomics (n = 3 donors per treatment group). (A) Principal-component analyses plot of lipid abundance in different treatment groups. (B) Abundance of phosphatidylcholine (PC), phosphatidylserine (PS), phosphatidylinositol (PI), and ceramides (Cer) as molar percentage of all lipids, depicted per treatment group. Data are represented as mean ± SEM. ***p < 0.001, **p < 0.01 between HKCA and HKCA + Cer1; ###p < 0.001 between HKCA and HKCA + Rapa; p values were calculated using a one-way ANOVA with Dunnett’s post-test. (C) Heatmap of lipid subspecies of PCs, PSs, PIs, and Cers. (D) Ratio of ceramide species containing unsaturated (one or two double bonds) versus saturated (no double bonds) fatty acid chains. ***p < 0.001; p values were calculated using a one-way ANOVA with Dunnett’s post-test. See also Figure S9.

In addition to the effect on cell metabolism, we explored if acid ceramidase inhibition affects the cellular lipidome. Previous studies indicate an important role of lipid metabolism in trained immunity and we observed before that trained immunity is accompanied by profound changes in the lipidome of monocytes.^3,18,22^ We analyzed the lipidome of isolated monocytes 24 h after stimulation with HKCA only, or in combination with ceranib-1 or rapamycin by quantitative shotgun lipidomics, an unbiased mass-spectrometry-based method that can identify hundreds of lipid species in cells.^23^ Principal-component analysis of the obtained data revealed substantial differences between the lipidomes of HKCA-trained cells and those treated with HKCA and ceranib-1 (Figure 4A). Ceranib-1 increased the abundance of phosphatidylcholines (PCs), whereas it reduced the concentration of phosphatidylserines (PSs) and phosphatidylinositols (PIs) (Figure 4B) compared with HKCA-treated samples. The effect of ceranib-1 on these lipid classes mirrored the lipidome of untrained monocytes and those treated with rapamycin, indicating that acid ceramidase inhibition prevents the lipidome characteristic of trained immunity. A similar pattern emerged at the level of subspecies of PC, PS, and PI, with rapamycin and ceranib-1 treatment opposing the effects of HKCA training (Figures 4C and S9A–S9C). As expected, ceranib-1 treatment markedly increased nearly all measured ceramide species (Figures 4C and S9D). No differences in ceramide levels were observed between the untrained, HKCA-trained and rapamycin-treated cells. In addition to the changes in concentration of ceramide species, the fatty acid saturation of ceramides were altered by acid ceramidase inhibition. Treatment with ceranib-1 resulted in a lower ratio of unsaturated ceramide species to saturated ceramide species (Figure 4D).

Together, our data indicate that trained immunity is associated with distinct changes in the lipidome, most notably in PCs, PSs, and PIs. These effects are reversed by both acid ceramidase and mTOR inhibition.

### Acid ceramidase inhibition alters metabolic and histone-modifying gene expression

**Figure 5.**
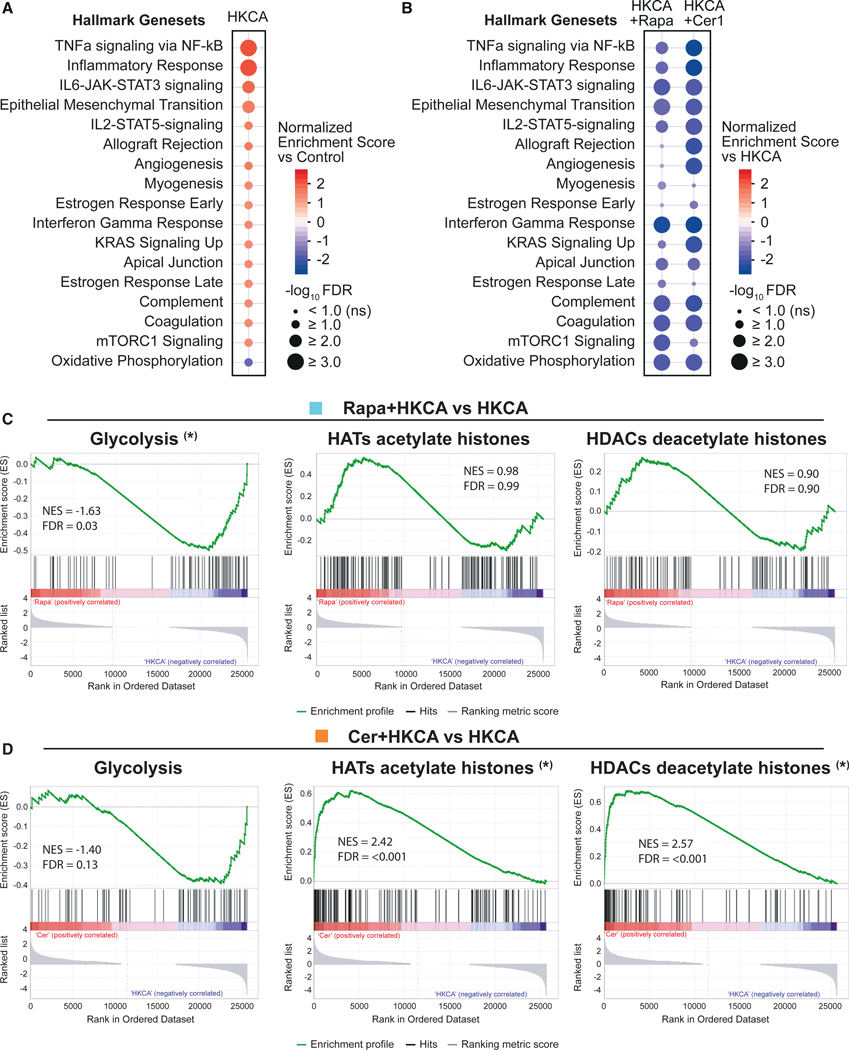
Acid ceramidase inhibition affects monocyte gene transcription (A–D) Transcriptome analysis in primary human monocytes on day 1 after HKCA training with or without rapamycin (Rapa) or ceranib-1 (Cer1) treatment (n=3 donors per group). (A and B) Differentially expressed gene sets of the Hallmark database in HKCA-treated monocytes versus RPMI-treated controls (A) (false discovery rate [FDR] < 0.1) and differential expression of these gene sets in monocytes trained with HKCA and treated with rapamycin or ceranib-1 versus HKCA-trained cells (B). (C and D) Gene set enrichment analysis of HKCA + rapamycin versus HKCA dataset (C) and HKCA + ceranib-1 versus HKCA dataset (D) for the reactome gene sets ‘‘Glycolysis,’’ ‘‘HATs acetylate histones,’’ and ‘‘HDACs deactylate histones.’’ Asterisks indicate gene sets with significant FDR. NES, normalized enrichment score; FDR, false discovery rate; HAT, histone acetyltransferase; HDAC, histone deactylase; INF, infinity. See also Figure S10.

We were interested in investigating the effect of acid ceramidase inhibition on gene transcription in HKCA-trained monocytes and compare it with the effects of mTOR inhibition. For this purpose, PBMCs were stimulated for 24 h with HKCA and treated with RPMI (negative control), rapamycin, or ceranib-1. Subsequently, we purified monocytes and performed RNA sequencing. We found 3,967 differentially expressed genes in cells treated with HKCA + ceranib-1 compared with HKCA only and identified 1,863 differentially expressed genes in cells treated with HKCA + rapamycin compared with HKCA only (log2 fold change[FC] > 1.00, false discovery rate < 0.05). Gene set enrichment analysis (GSEA) using the Molecular Signatures Database (MSigDB) Hallmark gene set collection revealed that various genes involved in inflammation and immune cell functions are upregulated by HKCA training (Figure 5A). These same gene sets were downregulated in HKCA-trained monocytes treated with ceranib-1. This inhibitory effect on inflammatory gene transcription was similar in rapamycin-treated cells (Figure 5B).

Thereafter, we studied the effect of acid ceramidase inhibition on the expression of genes involved in cell metabolism and histone modification and compared this with the effects of mTOR inhibition. For this purpose, GSEA was performed on 13 gene sets of the REACTOME database. We observed that ceranib-1 had a modest effect on metabolic gene sets, while rapamycin markedly downregulated genes involved in cell metabolism (Figures 5C and 5D; Figures S10A and S10B, left panels). Ceranib-1 did have a pronounced effect on genes involved in histone acetylation and deacetylation as well as histone methylation and demethylation, while rapamycin did not affect these gene sets (Figures 5C and 5D; Figures S10A and S10B, middle and right panels).

These data show a clear impact of acid ceramidase inhibition on genes related to histone modifications, indicating a direct link between acid ceramidase and epigenetic regulation in monocytes.

### Acid ceramidase inhibition induces histone modifications

**Figure 6.**
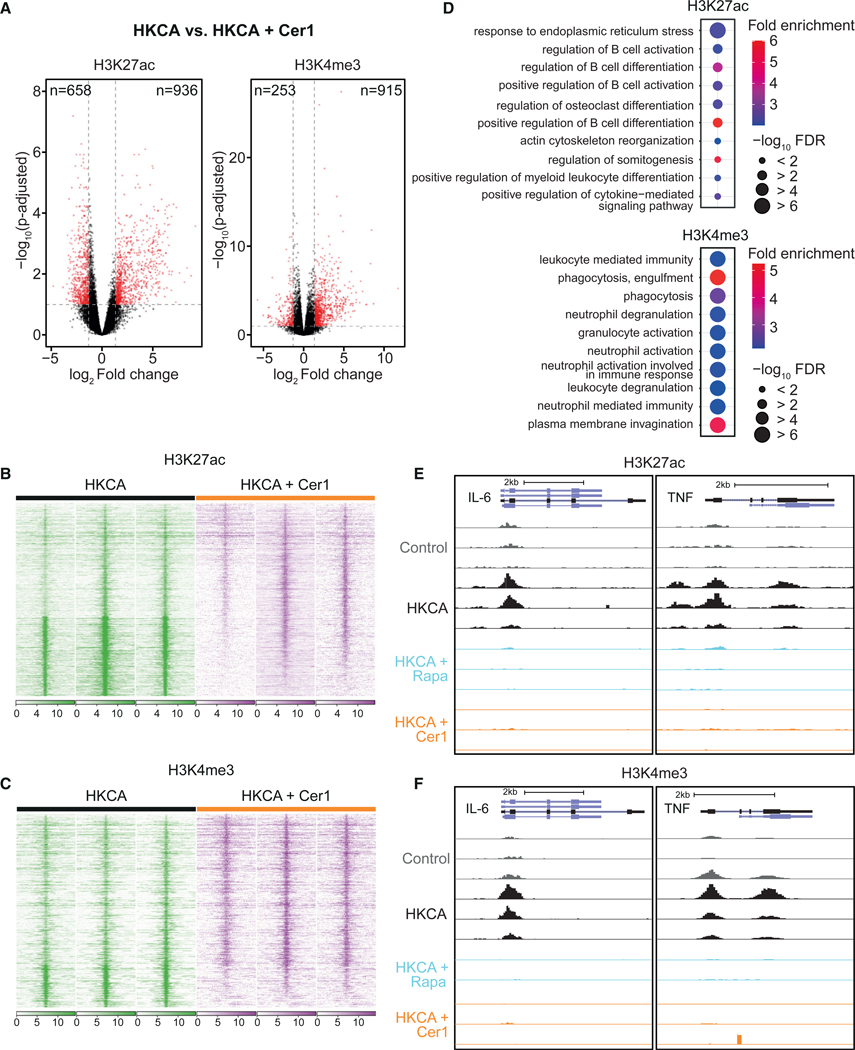
Inhibiting acid ceramidase modulates the epigenetic configuration of monocytes (A–F) PBMCs were stimulated for 24 h with RPMI, HKCA, HKCA + ceranib-1 (Cer1), or HKCA + rapamycin (Rapa), after which the cells were rested for 5 days. The monocytes were subsequently purified and chromatin immunoprecipitation (ChIP) performed for H3K27ac and H3K4me3 (n = donors 3 per treatment group). (A) Volcano plot showing chromatin mark peaks with increased or decreased H3K27ac and H3K4me3 in cells treated with HKCA + ceranib-1 compared with HKCA alone. Cutoffs for significance was chosen at p-adjusted < 0.1 and fold change > 2.5. (B and C) Heatmaps showing the intensity (RPKM) of H3K27ac (B) and H3K4me3 (C) signals in a 10 kilobase window around significantly dynamic regions (fold change > 2.5, p < 0.1). (D) Top 10 gene ontology biological processes associated with genes located in proximity to dynamic ChIP-seq peaks in HKCA + ceranib-1 compared with HKCA-trained (fold change > 2.5, FDR < 0.1). (E and F) H3K27ac signal (E) and H3K4me3 signal (F) at IL-6 and TNF genes as visualized in the UCSC genome browser. RPKM, reads per kilobase of transcript per million mapped reads. See also Figure S11.

Histone 3 lysine 4 trimethylation (H3K4me3) and histone 3 lysine 27 acetylation (H3K27ac) play an important role in the epigenetic regulation of trained immunity in innate immune cells.^2^ Therefore, we investigated the effect of acid ceramidase inhibition on H3K4me3 and H3K27ac in HKCA-trained monocytes and compared it with mTOR inhibition. We performed a whole-genome assessment of H3K27ac and H3K4me3 by chromatin immunoprecipitation sequencing (ChIP-seq) in HKCA-trained monocytes treated with either ceranib-1 or rapamycin. Monocytes were trained as described above and, after 5 days of rest, monocyte-derived macrophages were harvested for ChIP-seq. We found that ceranib-1-treated HKCA-trained cells showed a substantially different epigenetic landscape compared with untreated HKCA-trained cells (Figure 6A). Nine hundred and thirty-six H3K27ac-peaks were induced and 658 reduced, and for H3K4me3 915 peaks were induced and 253 reduced in ceranib-1-treated cells (FC > 2.5, p < 0.1; Figures 6A–6C, S11A, and S11B). Rapamycin-treated cells showed fewer changes in H3K27ac and H3K4me3, with 228 H3K27ac peaks induced and 641 reduced, and 510 H3K4me3 peaks induced and 163 reduced compared with HKCA-trained cells (FC > 2.5, p < 0.1; Figures S11A and S11B). Pathway analysis of differentially regulated H3K27ac and H3K4me3 peaks in ceranib-1-treated cells versus HKCA-trained cells, using the Genomic Regions of Annotations Tool, revealed differently regulated pathways associated with immune responses (Figure 6D). In rapamycin-treated cells, we also revealed differently regulated pathways associated with immune responses and those associated with metabolism (Figures S11C and S11E). Both ceranib-1 and rapamycin treatment completely eradicated H3K27ac (Figures 6E and S11D) and H3K4me3 (Figures 6F and S11F) marks around the promoter of IL-6 and TNF.

These data demonstrate that inhibition of acid ceramidase has a profound effect on the epigenetic landscape of monocytes and thereby modulates the accessibility of inflammatory genes for transcription. Whether the epigenetic changes are a result of the above-described transcriptional changes in histone-modifying enzymes needs to be further elucidated in further studies. The epigenetic changes mediated by acid ceramidase inhibition include a broader set of genomic regions compared with those associated with mTOR inhibition.

### Single-nucleotide polymorphisms around the gene encoding acid ceramidase (*ASAH1*) associate with trained immunity

**Figure 7.**
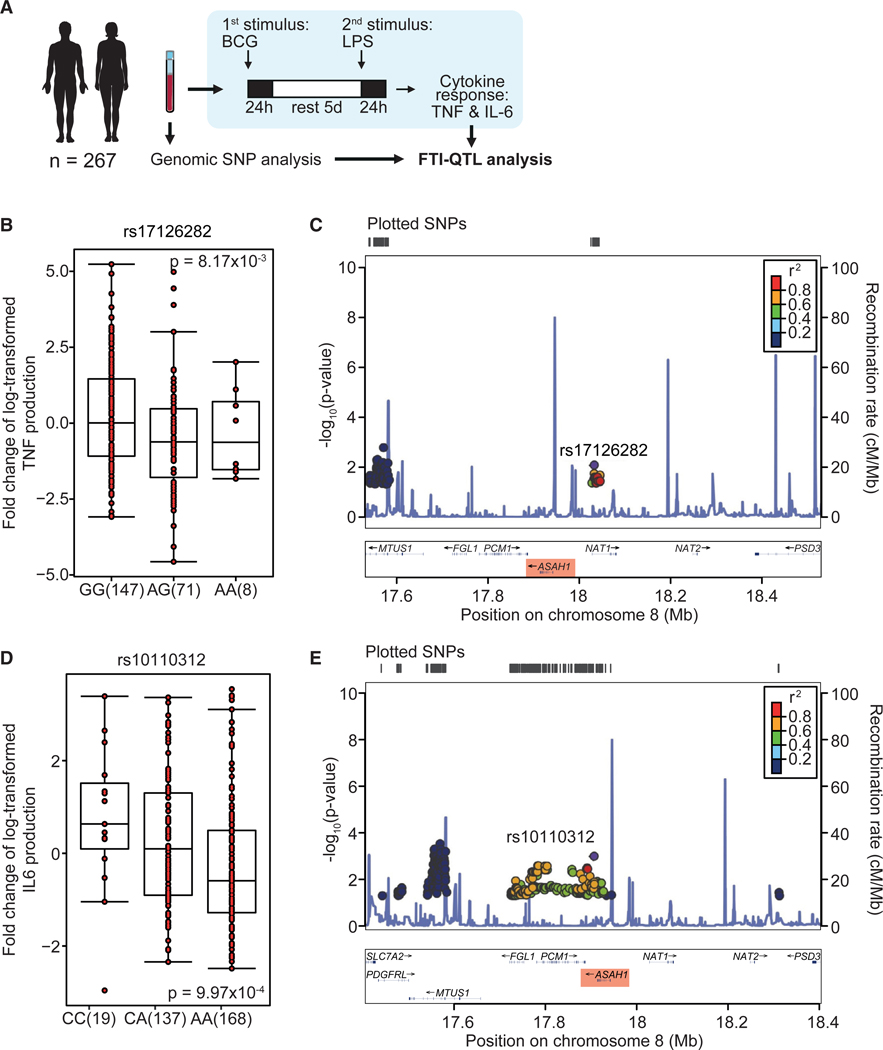
Single-nucleotide polymorphisms in the proximity of *ASAH1* associate with trained immunity cytokine responses (A) Schematic representation of our *in vitro* training experiments combined with QTL analysis using single-nucleotide polymorphism (SNP) genotypes of 267 volunteers from the 300BCG cohort. (B–E) Associations of SNPs rs17126282 and rs10110312 near *ASAH1* with the cytokine responses following training with Bacillus Calmette-Guérin (BCG) vaccine. Boxplots show the genotype stratified fold changes in TNF and IL-6 responses for SNPs rs17126282 (B) (n = 238 donors, p = 8.17 × 10^−3^) and rs10110312 (D) (n = 251 donors, p = 9.97 × 10^−4^), respectively. (C and E) Regional association plots around SNPs rs17126282 (C) and rs10110312 (E) that were identified to be associated with cytokine changes upon *in vitro* training. SNPs are plotted as –log10 of the p values. Estimated recombination rates are shown in blue to reflect the local LD structure (based on the CEU population). The correlation of the lead SNP (purple) to other SNPs (p < 0.05) is indicated by color (with red indicating highly correlated and blue indicating weakly correlated). The fold change in cytokine production between RPMI and LPS restimulated cells was mapped to genotype data using a linear regression model with age and sex as covariates. FTI-QTL, functional trained immunity quantitative trait locus. See also Figure S12.

To confirm acid ceramidase’s role in regulating trained immunity, we conducted an FTI-QTL analysis using the genotype data and cytokine measurements of human monocytes isolated from 267 healthy adults from the Netherlands belonging to the 300BCG cohort of the Human Functional Genomics Project (Figure 7A).^13^ Fifty-six percent of participants were females and the age range was 18–71 years. DNA samples of all 267 individuals were genotyped to identify single-nucleotide polymorphisms (SNPs) and monocytes of the same individuals were stimulated with Bacillus Guérin-Calmette (BCG) to induce trained immunity, which was assessed by the TNF and IL-6 cytokine production upon LPS restimulation 5 days after BCG training (Figure 7A). The FTI-QTL analysis identified two SNPs in the proximity of *ASAH1* (in a window of 150 kilobases), the gene encoding acid ceramidase, showing a significant association with the capacity of BCG-induced trained immunity. SNPs rs17126282 and rs10110312 were associated with the trained cytokine response of TNF (n = 238, p = 8.17 3 10^−3^) and IL-6 (n = 251, p = 9.97 3 10^−4^), respectively (Figures 7B–7E). FTI-QTL also revealed QTL effects in other genes involved in sphingolipid metabolism. We identified SNPs in the proximity of *ASAH2*, *CERK*, *CERS2*, *4*, *5*, and *6*, *PLPP1*, *2*, and *3*, *SGPP2*, and *SPHK2* to be associated with trained immunity-induced cytokine responses (Figure S12). Together, these data demonstrate the importance of acid ceramidase, and other sphingolipid-relevant enzymes, in regulating trained immunity.

## DISCUSSION

Sphingolipids are implicated in a wide range of cellular functions and play key roles in inflammation.^15^ Here, we reveal the role of sphingolipids in regulating trained immunity. We show that exogenously administered sphingolipids or inhibition of enzymes involved in sphingolipid metabolism modulate trained immunity. We found that inhibiting acid ceramidase, an enzyme that converts ceramide to sphingosine, potently suppresses the induction of trained immunity. This is accompanied by a modified cell metabolism and lipidome. The functional and metabolic changes resulting from acid ceramidase inhibition are comparable with those induced by inhibiting mTOR, a known regulator of trained immunity.^10^ Despite these apparent similarities, we also revealed an important difference, namely that acid ceramidase changes the expression of genes involved in histone modifications. As a consequence, acid ceramidase inhibition results in profound reprogramming of the epigenetic landscape of monocytes, including histone modifications of genes involved in inflammation. We validated our finding by performing an FTI-QTL analysis in healthy adults from the Human Functional Genomics Project and found two SNPs in the proximity of *ASAH1*, the gene encoding acid ceramidase, to be associated with the trained immunity cytokine response.

In humans, five different ceramidases have been identified, including one neutral ceramidase (with three identified isotypes), three alkaline ceramidases, and one acid ceramidase. The ceramidases differ in their intracellular location, structure and function, and pH dependency.^24^ Acid ceramidase is most abundant in the lysosomal compartment, where it hydrolyzes ceramides into sphingosine and a free fatty acid at a pH of ~4.5.^25^ Acid ceramidase requires saposin D to access its substrate and perform its enzymatic activity.^26,27^ We recently described that prosaposin, the precursor protein of saposins A to D, has important immunoregulatory functions.^21^ Genetic mutations in the gene encoding for acid ceramidase (*ASAH1*) can cause two rare lysosomal storage diseases depending on the type of mutation, namely Farber’s disease and spinal muscular atrophy with progressive myoclonic epilepsy.^28–30^ Farber’s disease is a lysosomal storage disorder characterized by psychomotor deterioration and macrophage-driven inflammation causing arthritis, subcutaneous granuloma formation, and histiocytosis of multiple organs, including liver, spleen, and lymph nodes, which illustrates acid ceramidase’s crucial role in regulating innate immune responses.^31,32^ There is growing evidence of acid ceramidase involvement in the pathogenesis of other innate immune cell-mediated diseases, including Alzheimer’s disease, cancer, type 2 diabetes, inflammatory bowel disease, and infectious diseases.^33–40^

In this study, we showed that blocking acid ceramidase drastically alters cell metabolism. The metabolic reprogramming may be partly established by modulating mTOR signaling and corroborates previous observations showing a link between acid ceramidase and mTOR activity.^41^ Furthermore, inhibiting acid ceramidase leads to ceramide accumulation, which can directly affect cell metabolism by inhibiting uptake of glucose and amino acids and stimulating fatty acid utilization for energy production.^42–45^ Ceramides also affect mitochondrial activity by changing the mitochondrial membrane potential and respiratory chain activity.^46–48^ Delivery of ceramides to monocytes via sphingolipid-loaded NB, however, does not lead to profound effects on trained immunity responses. Therefore, inhibition of acid ceramidase might not directly impact trained immunity through increased ceramide levels. We also observed that acid ceramidase inhibition has marked effects on monocytes’ lipid composition. Our lipidomic studies show that HKCA training decreases PCs, while PSs and PIs are increased. Changes in PCs, PSs, and PIs are reverted by acid ceramidase inhibition and may reflect changes in cell and organelle membranes, which could affect the function of membrane receptors and their downstream signaling.^49^ For example, mTOR activation by the PI3K/AKT pathway depends on PS, which brings AKT to the plasma membrane where it can be activated by PIs.^50,51^ Inhibition of acid ceramidase also led to an increase in ceramide species with saturated fatty acid chains. In addition, delivery of sphingolipid species with a saturated or unsaturated fatty acid chain resulted in opposite effects on the cytokine response after HKCA training, highlighting the influence of lipid saturation levels in controlling trained immunity. The saturation level of ceramides influences the formation of tightly packed ceramide-rich membrane domains that influence membrane fluidity and the activity of membrane-bound receptors.^52–55^ Ceramide-rich membrane domains are thought to contain high concentrations of membrane-bound receptors forming cellular signaling hubs. Introduction of double bonds in the ceramide acyl chain disrupts the formation of these tightly packed ceramide clusters, since unsaturated ceramides require more lateral space, making it more difficult to form tight connections with neighboring ceramides. The saturation level of ceramides has been shown to have the greatest effect on membrane order, larger than varying headgroups, acyl chain length, or hydroxylation of ceramides.^56^

Although we found a clear link between sphingolipid metabolism and mTOR signaling, our data indicate that this cannot fully explain the epigenetic modifications induced by acid ceramidase inhibition. We observed that acid ceramidase inhibition affects the transcription of genes related to histone modifications, whereas mTOR inhibition does not. The changes in H3K4me3 and H3K27ac induced by inhibition of acid ceramidase were also more extensive compared with mTOR inhibition. The question arises how acid ceramidase then mediates these epigenetic effects. One possible answer is that inhibition of acid ceramidase causes a reduction in S1P. S1Ps can signal in a paracrine/autocrine manner via one of its five G-protein-coupled receptors, S1PR1–5, on the plasma membrane or through intracellular pathways.^57^ S1Ps are known to be involved in TLR activation via TNF receptor-associated factors, leading to the activation of the pro-inflammatory transcription factor nuclear factor κβ (NF-κβ), which mediates extensive chromatin remodeling.^58,59^ Furthermore, S1P is also directly linked to epigenetic regulation. In the nucleus, S1P binds to and inhibits histone deacetylases 1 and 2 (HDAC1 and HDAC2), leading to the decondensation of chromatin and initiation of transcription.^60^ Another possible answer is that inhibition of acid ceramidase causes an increase in ceramide-1-phosphate (C1P). C1P also resides in the nucleus. Although its precise nuclear function is currently unknown, C1P could be involved in epigenetic regulation.^61,62^ Previous studies showed that C1P exerts anti-inflammatory effects, reducing cytokine responses triggered by exposure to LPS.^63^ We found that exogenous administration of C1P inhibits trained immunity, and that inhibition of the enzyme required for production of C1P (with NVP231) augments trained immunity. Acid ceramidase itself can also be found in the nucleus and could also be directly involved in epigenetic regulation.^61^ This is supported by the fact that acid ceramidase interacts with SET domain bifurcated histone methyltransferase 1, a protein involved in epigenetic regulation.^64^

Modulating trained immunity has therapeutic potential in a broad range of diseases, including infectious diseases, autoinflammatory disorders, cancer, atherosclerosis, and organ transplantations.^5–7,9^ Our data show that acid ceramidase, and ceramide metabolism, represent promising therapeutic targets that can be explored to control trained immunity. A variety of inhibitors of acid ceramidase have been developed (reviewed in Saied and Arenz^65^), of which the most potent are the small-molecule inhibitors ceranib-1/2, carmofur, compound 17a, and compound E2.^66–69^ The widely used anti-estrogen drug tamoxifen also inhibits acid ceramidase.^70^ Another therapeutic strategy is the direct delivery of sphingolipids using sphingolipid-containing nanotherapeutics. We demonstrated the feasibility of this approach by developing a library of sphingolipid-loaded nanobiologics that inherently engage myeloid cells and potently modulate their inflammatory response.

Collectively, our findings advance the insight into how trained immunity is regulated. We show that sphingolipids modulate trained immunity. In particular, we found that the sphingolipid-metabolizing enzyme acid ceramidase is a key regulator of trained immunity as well as the associated metabolic and lipidomic reprogramming. Furthermore, we reveal that acid ceramidase determines the expression of histone-regulating genes, thereby reshaping the epigenetic landscape of monocytes. Our data identify sphingolipid metabolism, and acid ceramidase in particular, as a potential therapeutic target for treating disorders in which trained immunity is involved.

### Limitations of the study

We investigated the effect of acid ceramidase on trained immunity in an *in vitro* model using primary human monocytes trained with HKCA. A next step would be to confirm that inhibition of acid ceramidase can also suppress trained immunity induced by other infectious (e.g., BCG) or non-infectious stimuli (e.g., pro-inflammatory cytokines such as IL-1β, or disease-specific stimuli such as oxidized low-density lipoprotein in atherosclerosis). The results presented in this study are for an important part based on inhibiting enzymes of the sphingolipid pathway using small-molecule inhibitors. Although the inhibitors used are specific and potent in inhibiting the different sphingolipid-converting enzymes, and we could reproduce results by using different small-molecule inhibitors, we cannot fully exclude off-target effects of the small-molecule inhibitors. Our *in vitro* data convincingly show that ceranib-1 suppresses trained immunity at the functional, metabolic, transcriptional, and epigenetic level. Future studies, in *in vivo* experimental models and in humans, should determine if suppression of trained immunity by inhibition of acid ceramidase is effective and safe for treating disorders in which trained immunity is involved.

## STAR★METHODS

### RESOURCE AVAILABILITY

#### Lead contact

Further information and requests for resources and reagents should be directed to and will be fulfilled by the Lead Contact, Raphaël Duivenvoorden (Raphael.Duivenvoorden@radboudumc.nl).

#### Materials availability

Materials are available upon reasonable request from the lead contact.

#### Data and code availability

- The RNA-seq and ChIP-seq datasets generated during this study are publicly available at GEO under accession number GSE188641.
- This paper does not report original code.
- Any additional information required to reanalyze the data reported in this paper is available from the lead contact upon request.

### EXPERIMENTAL MODEL AND STUDY PARTICIPANT DETAILS

#### Human subjects

For *in vitro* studies on human PBMCs and monocytes, buffy coats from healthy donors were obtained from Sanquin blood bank, Nijmegen, after written informed consent, from which no additional details are available.

#### Human 300BCG cohort

The 300BCG cohort consists of 267 healthy males and females of Western European ancestry. The 300BCG cohort study was approved by the local ethics committee (CMO regio Arnhem-Nijmegen, number NL58553.091.16). Inclusion of volunteers and experiments were conducted according to the principles expressed in the Declaration of Helsinki. All volunteers gave written informed consent before any material was taken.

### METHOD DETAILS

#### Human PBMC isolation

PBMCs were isolated by differential centrifugation over Ficoll-Paque (Lymphoprep, StemCell Technologies, Inc.). Cells were washed two times in PBS. PBMCs were resuspended in RPMI culture medium supplemented with 2 mM glutamax, 1 mM pyruvate and penicillin/streptomycin (all from Thermo Fisher Scientific) and counted on a Casy counter (Innovatis).

#### Training and inhibition experiments

Human PBMCs were trained as described before.^18^ In short, 500.000 PBMCs were added into 96-well flat-bottom plates. Cells were allowed to adhere for 1 h at 37°C. Cells were washed three times with PBS prior to stimulations. After washing cells were incubated with culture medium only as negative control, or treated with 0.1 μM rapamycin (Seleckchem), 10 μM ceranib-1 (R&D Systems), 1 μM NVP231 (R&D Systems), 1 μM SLM6031434 (Sigma Aldrich), 1 μM PF543 (Sigma Aldrich), 500 μM Fumonisin B1 (Sanbio), 50 μM Carmofur (Merck life Science) or 50 μM ARN14974 (Sanbio) for 1 h at 37°C. Subsequently, cells were incubated with 10^5^ cells/ml HKCA (Invivogen) together with the respective treatment for 24 h at 37°C. Next, cells were washed and cells were rested for five days in RPMI culture medium containing 10% FBS (Serana Europe GmbH). After the resting period cells were stimulated with either RPMI as negative control, 10 ng/mL LPS (O55:B5, Invivogen) or 1 μg/mL Pam3CSK4 (Invivogen).

#### Lactate dehydrogenase measurements to assess cellular toxicity

LDH concentration was measured in supernatants of PBMCs after 24-h incubation with small molecule inhibitors or nanoparticles using CyQuant LDH Cytotoxicity Assay (Thermo Fisher Scientific). LDH concentration is calculated as percentage of maximal possible LDH concentration in completely lysed cells according to formula: $LDH(\%\;of\;\max)=(\frac{Compound\;\text{induced}\;LDH-Spontaneous\;LDH}{Maximum\;LDH-Spontaneous\;LDH})\times 100$.

#### Western blotting of human PBMC lysates

Human PBMCs were incubated with culture medium only as negative control, or treated with 10 μM ceranib-1 (R&D Systems) or 0.1 μM rapamycin (Seleckchem) for 1 h at 37°C. Subsequently, cells were treated with 10^5^ cells/ml HKCA (Invivogen) together with the respective treatment for 24 h at 37°C. Cells were washed with PBS and scraped in lysis buffer containing 50 mM HEPES Buffer pH 7.0, 250 mM sodium chloride, 0.1% NP-40, 5 mM EDTA, 0.5 mM dithiothreitol (DTT) and proteinase inhibitor cocktail (Roche). Protein content was measured with μBCA kit (Thermo Fisher Scientific) according to manufacturer’s instructions. Equal amounts of total protein were prepared in Laemmli sample buffer (Bio-rad) and resolved by SDS-PAGE and subsequently transferred onto nitrocellulose blotting membranes (GE Healthcare). Blots were blocked using blocking reagent (Roche) and incubated with S6K1 antibodies (both Cell Signaling) followed by an appropriate peroxidase-labeled secondary antibody (Jackson Immuno Research). Blots were developed using WesternBright Quantum detection kit (Advansta) according to manufacturer’s instructions.

#### Seahorse experiments

Human PBMCs were incubated with culture medium only as negative control, or treated with 10 μM ceranib-1 (R&D Systems) or 0.1 μM rapamycin (Selckchem) for 1 h at 37°C. Subsequently, cells were treated with 10^5^ cells/ml HKCA (Invivogen) together with the inhibitors for 24 h at 37°C. Cells were washed and cells were rested for five days in RPMI culture medium containing 10% FBS. Subsequently, cells were washed with PBS and incubated with versene solution (0.48 mM EDTA, Sigma Aldrich) for 30 min at 37°C. Cells were scraped from the plates and seeded in an XF96 microplate (Agilent Technologies) coated with 1.98 mg/mL in 5% acetic acid Corning Cell-Tak Cell and Tissue Adhesive (BD Biosciences). 300.000 cells per well were seeded in quintuple in RPMI 1640 medium and let to adhere for approximately 30 min at 37°C and 5% CO_2_. Next, the RPMI was removed and replaced by nonbuffered DMEM without glucose (Sigma Aldrich), supplemented appropriately for each metabolic assay with 0 mM or 11 mM D-Glucose (Sigma Aldrich), 0 mM or 1 mM Pyruvate (Sigma Aldrich) and 1 mM or 2 mM L-Glutamine (Sigma Aldrich). Thereafter, cells were kept in a CO_2_-free incubator (37°C) for 45–60 min followed by measurements of oxygen consumption and extracellular acidification. These measurements were performed at 37°C using a Seahorse XF96 Extracellular Flux Analyzer (Agilent Technologies). The Seahorse XF Cell Glyco Stress Test was performed to determine the dynamics of the glycolytic rate based on the extracellular acidification rate (ECAR). The cells were treated with glucose (11 mM), oligomycin (1 μM; Sigma Aldrich) and 2-Deoxy-D-Glucose (22 mM; Sigma Aldrich) respectively at timepoints indicated in Figure 3B. To determine the dynamics in mitochondrial oxygen consumption rate (OCR), the Seahorse XF Cell Mito Stress Test was performed. During this test, cells were treated with oligomycin (1 μM; Sigma-Aldrich), Carbonyl cyanide-4-(trifluoromethoxy)phenylhydrazone (FCCP; 1 μM; Sigma Aldrich) and a combination of rotenone (1.25 μM; Sigma Aldrich) and antimycin A (2.5 μM; Sigma Aldrich) at timepoints indicated in Figure 3A.

#### Formulating sphingolipid-loaded nanobiologics

1,2-dimyristoyl-*sn*-glycero-3-phosphocholine (DMPC), cholesterol and sphingolipids were obtained from Avanti Lipids with a purities >99%. ApoA-1 was isolated from human HDL concentrate (Biosource Technology) as previously reported by us.^9^ DMPC 1.00 eq., 3.69 umol), cholesterol 0.20 eq., 0.74 umol) en sphingolipid 0.25 eq., 0.92 umol).were placed in a 20 mL vial and dissolved in chloroform (2.0 mL). For the unloaded-nanobiologics, used as controls, the sphingolipids were substituted by an additional 0.2 eq. DMPC. The solvent was evaporated under vacuum to create a lipid film, followed by the addition of apoA-1 (1.0 mg, 33 nM) in PBS (5.0 mL). The suspension was sonicated using a Branson Digital Sonifier SFX150 working at 60% power output for 7 min while being cooled in an ice-water bath. The slightly opaque solution was concentrated by centrifugal filtration using Vivaspin tubes (Sartorius Biotech, 10 kDa molecular weight cut-off at 4000 rpm and 4°C) until a volume of approximately 1.0 mL remained. PBS (2.0 mL) was added and the sample again centrifuged until 1.0 mL remained; this was repeated once more. The resulting solution was filtered through a 0.22 mm polyethersulfone (PES) syringe filter (Celltreat) to obtain the sphingolipids-loaded nanobiologics as a white emulsion.

#### Characterizing the size and sphingolipid-loading of the sphingolipid-loaded nanobiologics

Particle size was determined by dynamic light scattering (DLS) using a Brookhaven Instrument Corporation ZetaPALS analyzer. An aliquot (20 μL) of the nanobiologics was diluted with PBS (1mL) and filtered using a 0.22 μm PES syringe filter to remove any dust and micro-organisms. Six separate runs of 1 min each were recorded, and the mean of the number average size distribution reported. The sphingolipid concentration in the nanobiologic emulsions was determined by H NMR. An aliquot of the formulated sphingolipid-loaded nanobiologics (~0.25 mL) was freeze-dried and resuspended in a mixture of deuterated chloroform and methanol (50:50 vol.%, 0.5 mL total) containing a known concentration of DMPC. The sample was analyzed by H NMR and the intensities of signals characteristic of DMPC (CH_2_-COO) and sphingolipid (double bonds) used to determine the sphingolipid concentration.

#### Training experiments with sphingolipid-nanoparticles

Human PBMCs were isolated and plated as described above. After washing, cells were incubated with culture medium only as negative control, or treated with sphingolipid-loaded nanobiologics for 1 h at 37°C. Cells were then incubated with 10^5^ cells/ml HKCA (Invivogen) together with the respective sphingolipid-loaded nanoparticle (50 μM) for 24 h at 37°C. Subsequently, cells were washed, and rested for five days in RPMI culture medium containing 10% FBS. After the resting period cells were stimulated with either RPMI as negative control, 10 ng/mL LPS (Invivogen) or 1 μg/mL Pam3CSK4 (Invivogen).

#### Monocyte isolation

Monocytes were isolated using negative MACS isolation with the Pan monocyte isolation kit (Miltenyi Biotech). Briefly, stimulated PBMCs were washed with PBS and incubated with versene solution (0.48 mM EDTA, Sigma Aldrich) for 30 min at 37°C. Cells were scraped from the plates, counted, spun down and resuspended in MACS isolation buffer (PBS with 0.5% BSA and 2 mM EDTA). Monocyte isolation was performed according to manufacturer’s instructions.

#### Cytokine measurements

Cytokine production was measured in supernatants using commercial ELISA kits for human TNF, IL-6, IFNγ and IL-1β (R&D systems) according to manufacturer’s instruction.

#### RNA isolation, library preparation and sequencing for transcriptomic analysis

RNA-analysis was performed on monocytes isolated using MACS from PBMCs stimulated with the indicated conditions for 24 h. For RNA isolation 1*10^6^ isolated monocytes were resuspended in 350 μL of RNA later Buffer (QIAGEN). RNA was isolated using RNeasy kit (QIAGEN) including DNase I (QIAGEN) digestions. Total RNA isolated from monocytes was used for the preparation of the RNA sequencing libraries using the KAPA RNA HyperPrep Kit with RiboErase (KAPA Biosystems). In short, oligo hybridization and rRNA depletion, rRNA depletion cleanup, DNase digestion, DNase digestion cleanup, and RNA elution were performed according to protocol. Fragmentation and priming were performed at 94°C for 6 min. First strand synthesis, second strand synthesis and A-tailing were performed according to protocol. For the adaptor ligation, a 1.5 mM stock was used (NextFlex DNA barcodes, Bioo Scientific). First and second post-ligation cleanup was performed according to protocol. A total of 11 PCR cycles were performed for library amplification. The library amplification cleanup was done using a 0.8 x followed by a 1.0 x bead-based cleanup. Library size was determined using the High Sensitivity DNA bioanalyzer kit, and the library concentration was measured using the dsDNA High Sensitivity Assay (Denovix). Paired-end sequencing reads of 50 bp were generated using an Illumina NextSeq 500.

#### Flow cytometric analysis

After stimulations cell were scraped and dissolved in PBS containing 1% bovine serum albumin (BSA, Sigma). Cells were washed twice and incubated in 10% heat-inactivatedhuman serum (Serana) for blocking for 30 min on ice in dark. Antibody mixes for extracellular staining were added and incubated for 30 min on ice in dark. Antibodies used were anti-CD45-BV510 (Biolegend, 1:100, RRID: AB_2561383), anti-CD14-PE/Cyanine-7 (eBioscience, 1:100, RRID: AB_1582276), anti-CD16-FITC (eBioscience, 1:100, RRID: AB_10805747), anti-CD3-APCCy7 (Biolegend, 1:100, RRID: AB_2563410), and anti-CD11b-Alexa647 (Biolegend, 1:100, RRID: AB_493020), anti-CD80-BV421 (Biolegend, 1:100, RRID: AB_2564407), anti-HLA-DR-BV650 (Biolegend, 1:100, RRID: AB_2563828), anti-CD40-BV785 (Biolegend, 1:100, RRID: AB_2566211), live/dead stain FVS620 (BD Bioscience). 10% Brilliant Stain Buffer (BD Biosciences) was added to the antibody mix. Cells were resuspended in PBS 1% BSA and acquired with ACEA Novocyte 3000 (Agilent). For apoptosis and necrosis detection cells were stained with antibodies anti-CD45-eFluor405 (eBioscience, 1:100, RRID: AB_2637382), anti-CD16-APC (eBioscience, 1:100, RRID: AB_2016663) and anti-CD14PE-Cy7 (eBioscience, 1:100, RRID: AB_1582276) followed by staining with FITC Annexin V Apoptosis detection kit (Biolegend) according to manufacturer’s instruction.

#### Preparation of samples and lipid extraction for mass spectrometry lipidomics

Lipidomic analysis was performed on monocytes isolated using MCAS from trained PBMCs (RPMI, HKCA, HKCA + Cer1 or HKCA + Rapa) at day 6 after stimulations. For lipidomic analysis 10*10^6^ isolated monocytes were collected into microcentrifuge tubes, centrifuged at 1000 g for 5 min at 4°C. The supernatant was removed and cells were snap frozen in liquid nitrogen. Mass spectrometry-based lipid analysis was performed at Lipotype GmbH (Dresden, Germany) as described in Sampaio et al.^23^ Lipids were extracted using a two-step chloroform/methanol procedure.^83^ Samples were spiked with internal lipid standard mixture containing: cardiolipin 16:1/15:0/15:0/15:0 (CL), ceramide 18:1; 2/17:0 (Cer), diacylglycerol 17:0/17:0 (DAG), hexosylceramide 18:1; 2/12:0 (HexCer), lyso-phosphatidate 17:0 (LPA), lysophosphatidylcholine 12:0 (LPC), lyso-phosphatidylethanolamine 17:1 (LPE), lyso-phosphatidylglycerol 17:1 (LPG), lyso-phosphatidylinositol 17:1 (LPI), lyso-phosphatidylserine 17:1 (LPS), phosphatidate 17:0/17:0 (PA), phosphatidylcholine 17:0/17:0 (PC), phosphatidylethanolamine 17:0/17:0 (PE), phosphatidylglycerol 17:0/17:0 (PG), phosphatidylinositol 16:0/16:0 (PI), phosphatidylserine 17:0/17:0 (PS), cholesterol ester 20:0 (CE), sphingomyelin 18:1; 2/12:0; 0 (SM) and triacylglycerol 17:0/17:0/17:0 (TAG). After extraction, the organic phase was transferred to an infusion plate and dried in a speed vacuum concentrator. First step dry extract was resuspended in 7.5 mM ammonium acetate in chloroform/methanol/propanol (1:2:4, V:V:V) and second step dry extract in 33% ethanol solution of methylamine in chloroform/methanol (0.003:5:1; V:V:V). All liquid handling steps were performed using Hamilton Robotics STARlet robotic platform with the Anti Droplet Control feature for organic solvents pipetting.

#### Mass spectrometry data acquisition

Samples were analyzed by direct infusion on a QExactive mass spectrometer (Thermo Scientific) equipped with a TriVersa NanoMate ion source (Advion Biosciences). Samples were analyzed in both positive and negative ion modes with a resolution of Rm/z = 200 = 280000 for MS and Rm/z = 200 = 17500 for MSMS experiments, in a single acquisition. MSMS was triggered by an inclusion list encompassing corresponding MS mass ranges scanned in 1 Da increments.^84^ Both MS and MSMS data were combined to monitor CE, DAG and TAG ions as ammonium adducts; PC, PC O-, as acetate adducts; and CL, PA, PE, PE O-, PG, PI and PS as deprotonated anions. MS only was used to monitor LPA, LPE, LPE O-, LPI and LPS as deprotonated anions; Cer, HexCer, SM, LPC and LPC O- as acetate adduct.

#### Chromatin immunoprecipitation

Epigenetic analysis was performed on monocytes isolated using MACS from trained PBMCs (RPMI, HKCA, HKCA + Cer1 or HKCA + Rapa) at day 6 after stimulations. Isolated monocytes were resuspended in RPMI culture medium and fixed using formaldehyde (1% final concentration, Sigma Aldrich) for 10 min at room temperature. Unreacted formaldehyde was quenched with 125 mM glycine and incubated for 5 min at room temperature. Cells were washed twice in PBS containing protease inhibitor cocktail (Roche) and 1 mM PMSF (Roche), and subsequently, snap-frozen in liquid nitrogen. Cell pellets were stored at —80°C for further use. Cells were sonicated at a concentration of 15 million cells/ml using a Bioruptor pico sonicator (Diagenode; 10 cycles, 30 s on, 30 s off, at 4°C). Immunoprecipitation was performed using the MagnaChIP kit (Merck Millipore) according to manufacturer’s instruction. In short, 500,000 cells were incubated overnight with 1 mg H3K4me3 or H3K27Ac antibody (Diagenode) and protein A magnetic beads at 4°C. Beads and chromatin/antibody mixture were washed four times for 5 min at 4°C. After washing chromatin was eluted and proteins were degraded using proteinase K. DNA was purified using spin columns and eluted in Milli-Q water.

#### ChIP library preparation and sequencing

ChIP-seq libraries were prepared using the Kapa Hyper Prep Kit according to manufacturer’s protocol, with the following modifications. 2.5 mL of the NEXTflex adaptor stock (600 nM, Bioo Scientific) was used for adaptor ligation of each sample. Libraries were amplified with 12–15 PCR cycles followed by a double post-amplification clean-up was used to ensure proper removal of adapters. Samples were analyzed for purity using a High Sensitivity DNA Chip on a Bioanalyzer 2100 system (Agilent). Libraries were paired-end sequenced to a read length of 50 bp on an Illumina NextSeq500.

### QUANTIFICATION AND STATISTICAL ANALYSIS

#### *In vitro* experiment data analysis

For *in vitro* trainings-experiments, data are shown as mean ± SEM and significance is tested with one-way ANOVA with Dunnett’s post-test. Data were analyzed using GraphPad Prism 5.0. P-values of 0.05 were considered statistically significant. N numbers indicate the number of donors used per experiment.

#### Seahorse metabolic parameters analysis

The metabolic parameters depicted in Figure 3C were calculated from the OCR and ECAR (Figures 3A and 3B) as follows. The basal respiration was calculated as the average OCR before adding oligomycin. ATP-linked respiration was calculated as the average OCR of timepoints 1 to 4 subtracted by the average OCR of timepoints 5 to 7. The Proton leak was calculated as the average OCR of timepoints 5 to 7 subtracted by the average OCR of timepoints 11 to 13. The maximal respiration was calculated as the average OCR of timepoints 8 to 10 subtracted by the average OCR of timepoints 11 to 13. The spare respiratory capacity was calculated as the average OCR of timepoints 8 to 10 subtracted by the average OCR of timepoints 1 to 4. Non-mitochondrial oxygen consumption was calculated as the average OCR of timepoints 11 to 13. Glycolysis was calculated as the average ECAR of timepoints 5 to 7 subtracted by the average ECAR of timepoints 1 to 4. The glycolytic capacity was calculated as the average ECAR of timepoints 8 to 10 subtracted by the average ECAR of timepoints 1 to 4. Non-glycolytic acidification was calculated as the average ECAR of timepoints 1 to 4.

#### RNA-seq data analysis

RNA-sequencing reads were aligned with Hisat2 version 2.0.4 to the provided and pre-indexed hg38 transcript assembly from UCSC, with alignment tailoring for transcript assemblers enabled.^72^ Samtools was used to filter reads with a quality score lower than 20, and PCR duplicates were removed with Picard.^73^ Reads per gene were counted with the htseq-count script from the Hisat2 software suite using the GTF file corresponding to the transcript assembly, with reverse strandness enabled and identification attribute set to gene_id.

#### Gene set enrichment analysis

Gene set enrichment analyses (GSEA) were performed on normalized counts of 3 samples per treatment group using GSEA software v4.1.0 provided by Broad Institute.^77,85^ Genes for which no counts were detected across samples were excluded from analysis. To obtain a general overview of altered biological pathways between treatment groups, GSEA was performed using the 50 Hallmark gene sets of MSigDB as input. To investigate differences in metabolic and epigenetic pathways, GSEA was performed on a selection of 13 gene sets of the Reactome database. Analyses were conducted with 1000 gene set permutations and with the following settings: Metric for ranking genes: Signal2Noise; Remap/Collapse to gene symbols: Collapse; Enrichment statistic: weighted; Normalization mode: meandiv. For each gene set, a Normalized Enrichment Score (NES) was calculated. A gene set was considered enriched with a False Discovery Rate (FDR) < 0.10.

#### ChIP-seq data analysis

ChIP-sequencing data were aligned to human genome hg19 with BWA.^78^ Samtools was used to filter reads with a quality score lower than 20, and PCR duplicates were removed with Picard.^73^ Peaks were identified with MACS 2.2.6 in paired-end mode and ‘call-summits’ enabled at a false discovery rate of 0.01.^79^ A union of all identified peaks was generated with BEDTools, which was used to count reads per peak in each sample.^80^ Read counts were analyzed with DESeq2 to identify significant dynamics. We used GREAT to identify significantly associated gene ontologies, and to assign each ChIP peak to its closest gene.^81^ Heatmaps were generated using Fluff.^86^

#### Lipidomic data analysis

Data were analyzed with in-house developed lipid identification software based on LipidXplorer.^87,88^ Data post-processing and normalization were performed using an in-house developed data management system. Only lipid identifications with a signal-to-noise ratio >5, and a signal intensity 5-fold higher than in corresponding blank samples were considered for further data analysis.

Further data analysis was performed using a web-based analysis program lipotypeZoom. Figures were generated in R with the ggplot2 and ComplexHeatmap R packages.^71,75,76^ Data are shown as mean ± SEM, significance was determined using one-way ANOVA and Tukey post-test. For data representation of lipid species in heatmap (Figure 4C) only species, for which data of all three replicates in all conditions was available are depicted.

#### *In vitro* training in the 300BCG cohort and QTL mapping

We conducted *in vitro* BCG training of adherent PBMCs from 267 healthy individuals of Western European ancestry from the 300BCG cohort (NL58553.091.16) for whom we had both genotype and cytokine data. Adherent PBMCs were trained with BCG (5 μg/mL, BCG strain Bulgaria, Intervax) for 24 h, washed and rested for five days in normal culture medium. After the resting period, cells were stimulated with 10 ng/mL LPS (O55:B5, Sigma Aldrich). Cytokine production of TNFα and IL-6 was measured after 24 h in supernatant as described above. DNA samples of these individuals were genotyped using the commercially available SNP chip, Infinium Global Screening Array MD v1.0 from Illumina. Genotype information on approximately 4 million single-nucleotide polymorphisms (SNPs) was obtained upon imputation (MAF >5% and R^2^ > 0.3 for imputation quality). Genetic outliers (n = 17) were removed before QTL mapping. First, raw cytokine levels were log-transformed and the ratio between trained and non-trained cytokine levels taken as the change of cytokine levels. The cytokine changes were mapped to genotype data using a linear regression model with age and sex as covariates. R-package Matrix-eQTL was used for cytokine QTL mapping.^82^

#### Statistical analysis

Data are presented as mean ± SEM as not specified otherwise. The numbers of samples in each experiment are indicated as ‘n’ in the figure legends. For comparison of multiple conditions one-way ANOVA with Tukey’s or Dunnett’s post-test was used. Nlme and multcomp package in R or GraphPad Prism software (version 5) was used to calculate p values.^89,90^ To analyze the effect of genotype on the fold change cytokine production (Figures 7B and 7D) a linear regression model with age and sex as covariates was used.

## Supplementary Material

supplemental 1

## Figures and Tables

**Table:** KEY RESOURCES TABLE

REAGENT or RESOURCE	SOURCE	IDENTIFIER
Antibodies		

**RABBIT POLYCLONAL ANTI H3K4ME3**	Diagenode	Cat#pab-003-050; RRID: AB_2616052
**RABBIT POLYCLONAL ANTI-H3K27AC**	Diagenode	Cat#pab-196-050; RRID: AB_2637079
**DONKEY ANTI-RABBIT IGG (H+L) PEROXIDASE-LABELED**	Jackson Immuno Research	Cat#711-036-152
**RABBIT MAB ANTI-P70 S6 KINASE**	Cell Signaling	Cat#2708, RRID: AB_390722
**RABBIT MAB ANTI-PHOSPHO-P70 S6 KINASE**	Cell Signaling	Cat#9234, RRID: AB_2269803
**ANTI-CD45-BV510**	Biolegend	Cat#304035, RRID: AB_2561383
**ANTI-CD14-PE/CYANINE-7**	eBioscience	Cat#25-01490-42, RRID: AB_1582276
**ANTI-CD16-FITC**	eBioscience	Cat#11-0168-42, RRID: AB_10805747
**ANTI-CD3-APC/CYANINE-7**	Biolegend	Cat#300426, RRID: AB_2563410
**ANTI-CD11B-ALEXA647**	Biolegend	Cat#301319, RRID: AB_493020
**ANTI-CD80-BV421**	Biolegend	Cat#305222, RRID: AB_2564407
**ANTI-HLA-DR-BV650**	Biolegend	Cat#307650, RRID: AB_2563828
**ANTI-CD40-BV785**	Biolegend	Cat#334340, RRID: AB_2566211
**ANTI-CD45-EFLUOR405**	eBioscience	Cat#69-0459-42, RRID: AB_2637382
**ANTI-CD16-APC**	eBioscience	Cat#17-0168-42, RRID: AB_2016663

Biological samples		

**HUMAN PBMCS FROM BUFFY COATS**	Sanquin blood bank	Cat#B2825R00

Chemicals, peptide and recombinant proteins		

**FICOLL-PAQUE (LYMPHOPREP)**	StemCell Technologies, Inc.	Cat#07861
**GLUTAMAX**	Thermo Fisher Scientific	Cat#35050
**PYRUVATE**	Thermo Fisher Scientific	Cat#11360
**PENICILLIN/STREPTOMYCIN**	Thermo Fisher Scientific	Cat#15140
**RPMI**	Thermo Fisher Scientific	Cat#22409
**FETAL BOVINE SERUM**	Serana Europe GmbH	Cat#S-FBS-EU-025
**EDTA**	Sigma Aldrich	Cat#E5134
**DNASE I**	Qiagen	Cat#79254
**RAPAMYCIN (SIROLIMUS)**	Selleckchem	Cat#S1039
**HEAT-KILLED *CANDIDA ALBICANS***	Invivogen	Cat#tlrl-hkca
**LIPOPOLYSACCHARIDE (E.COLI, O55:B5), USED FOR *IN VITRO* TRAINING ON BUFFY COATS**	Invivogen	Cat#trlrl-pb5lps
**PAM3CSK4**	Invivogen	Cat#tlrl-pms
**CERANIB-1**	R&D Systems	Cat#4448
**NVP231**	R&D Systems	Cat#3960
**SLM6031434**	Sigma Aldrich	Cat#857381P
**PF543**	Sigma Aldrich	Cat#PZ0234
**FUMONISIN B1**	Sanbio	Cat#62580
**CARMOFUR**	Merck Life Science	Cat#C1494-10MG
**ARN14974**	Sanbio	Cat#17119-10
**HEPES BUFFER**	Sigma Aldrich	Cat#H3375
**NP-40**	Sigma Aldrich	Cat#I-3021
**DITHIOTHREITOL (DTT)**	Sigma-Aldrich	Cat#D9779
**16% FORMALDEHYDE**	Sigma Aldrich	Cat#28908
**PROTEASE INHIBITOR COCKTAIL (TABLETS)**	Roche	Cat#04693132001
**PHENYLMETHYLSULFONYL FLUORIDE (PMSF)**	Roche	Cat#11359061001
**NEXTFLEX DNA BARCODES**	Bioo Scientific	N/A
**NEXTFLEX ADAPTER STOCK**	Bioo Scientific	N/A
**LAEMMLI SAMPLE BUFFER**	Bio-rad	Cat#161-0737
**NITROCELLULOSE BLOTTING MEMBRANE**	GE Healthcare	Cat#10600002
**BRILLIANT STAIN BUFFER**	BD Bioscience	Cat#563794
**FVS620 LIVE/DEAD STAIN**	BD Bioscience	Cat#564996
**FITC ANNEXIN V APOPTOSIS DETECTION KIT**	Biolegend	Cat#640914
**BLOCKING REAGENT**	Roche	Cat#11526800
**SPHINGOSINE-1-PHOSPHATE (D18:1/16:0)**	Avanti Polar Lipids	Cat#860492
**SPHINGOSINE (D20:1)**	Avanti Polar Lipids	Cat#860660
**C24 CERAMIDE (D18:1/24:0)**	Avanti Polar Lipids	Cat#860524
**8C16 CERAMIDE (D18:1/16:0)**	Avanti Polar Lipids	Cat#860516
**C16 DIHYDROCERAMIDE (18:0/16:0)**	Avanti Polar Lipids	Cat#860634
**C24 DIHYDROCERAMIDE (D18:0/24:0)**	Avanti Polar Lipids	Cat#860628
**C16 CERAMIDE-1-PHOSPHATE (D18:1/16:0)**	Avanti Polar Lipids	Cat#860533
**C18:1 CERAMIDE-1-PHOSPHATE**	Avanti Polar Lipids	Cat#860599
**C12 CERAMIDE-1-PHOSPHATE**	Avanti Polar Lipids	Cat#860531
**C16 GALACTOSYL(A)CERAMIDE (D18:1/16:0)**	Avanti Polar Lipids	Cat#860431
**C16 GALACTOSYL(B)CERAMIDE (D18:1/16:A0)**	Avanti Polar Lipids	Cat#860521
**C24:0 GALACTOSYL(B)CERAMIDE (D18:1/24:0)**	Avanti Polar Lipids	Cat#860854
**C24:1 GALACTOSYL(B)CERAMIDE (D18:1/24:1)**	Avanti Polar Lipids	Cat#860546
**C24:0 LACTOSYL(B)CERAMIDE (D18:1/24:0)**	Avanti Polar Lipids	Cat#860577
**C24:1 LACTOSYL(B)CERAMIDE (D18:1/24:1)**	Avanti Polar Lipids	Cat#860597
**24:0 SPHINGOMYELIN**	Avanti Polar Lipids	Cat#860592
**24:1 SPHINGOMYELIN**	Avanti Polar Lipids	Cat#860593
**C18:0 GLUCOSYL(B)CERAMIDE (D18:1/18:0)**	Avanti Polar Lipids	Cat#860547
**C18:1 GLUCOSYL(B)CERAMIDE (D18:1/18:1(9Z))**	Avanti Polar Lipids	Cat#860548
**HUMAN HDL CONCENTRATE**	Bioresource Technology	N/A
**14:0 PC (DMPC)**	Avanti Polar Lipids	Cat#850345
**CHOLESTEROL**	Avanti Polar Lipids	Cat#700100
**CELLTAK**	BD Bioscience	Cat#734-1081
**DMEM**	Sigma Aldrich	Cat#D5030
**L-GLUTAMINE**	Sigma Aldrich	Cat#G3126
**SODIUM PYRUVATE**	Sigma Aldrich	Cat#P2256
**D-GLUCOSE**	Sigma Aldrich	Cat#G8644
**OLIGOMYCIN**	Sigma Aldrich	Cat#O4876
**ROTENONE**	Sigma Aldrich	Cat#R8875
**ANTIMYCIN A**	Sigma Aldrich	Cat#A8674
**CARBONYL CYANIDE-4-(TRIFLUOROMETHOXY)** **PHENYLHYDRAZONE (FCCP)**	Sigma Aldrich	Cat#C2920
**2-DEOXY-D-GLUCOSE**	Sigma Aldrich	Cat#D6134
**LIPOPOLYSACCHARIDE (*E*. COLI, O55:B5), USED IN THE 300BCG STUDY**	Sigma-Aldrich	Cat#L2880

Critical commercial assays		

**MACS PAN MONOCYTE ISOLATION KIT**	Miltenyi Biotech	Cat#130-096-537
**HUMAN IL-6 ELISA**	R&D systems	Cat#DY206
**HUMAN TNFA ELISA**	R&D systems	Cat#DY210
**HUMAN IFNG ELISA**	R&D systems	Cat#DY285B
**HUMAN IL-1SS ELISA**	R&D systems	Cat#DY201
**MBCA KIT**	Thermo Fisher Scientific	Cat#23235
**CYQUANT LDH CYTOTOXICITY ASSAY**	Thermo Fisher Scientific	Cat#C20301
**WESTERNBRIGHT QUANTUM KIT**	Advansta	Cat#K-12042-D10
**SLIDEFLASKS**	Thermo Fisher Scientific	Cat#170920
**RNEASY MINI KIT**	Qiagen	Cat#74106
**MAGNACHIP KIT**	Merck-Millipore	Cat#17-408
**KAPA RNA HYPERPREP KIT (WITH RIBOERASE)**	KAPA Biosystems	Cat#08098140702
**HIGH SENSITIVITY DNA BIOANALYZER KIT**	Agilent Technologies	Cat#5067-4626
**DSDNA HIGH SENSITIVITY ASSAY**	Denovix	N/A
**KAPA HYPER PREP KIT**	KAPA Biosystems	Cat#7962363001

Deposited data		

***IN VITRO* TRAINED MONOCYTE CHIP-SEQ AND RNA-SEQ DATA**	This paper	GEO: GSE188641

EXPERIMENTAL MODELS: ORGANISM/STRAINS		

**300BCG COHORT (HUMAN FUNCTIONAL GENOMICS PROJECT)**	N/A	https://www.humanfunctionalgenomics.org ^13^

Software and algorithms		

**GRAPHPAD PRISM**	Graphpad software	N/A
**LIPOTYPEZOOM**	Lipotype GmbH	N/A
**R**	R Core Team^71^	https://www.r-project.org/
**HISAT**	Kim et al.^72^	http://www.ccb.jhu.edu/software/hisat/index.shtml
**SAMTOOLS**	Li et al.^73^	http://samtools.sourceforge.net
**DESEQ2**	Love at al.^74^	http://www.bioconductor.org/packages/release/bioc/html/DESeq2.html
**GGPLOT2**	Wickham^75^	https://ggplot2.tidyverse.org/
**COMPLEX HEATMAPS**	Gu et al.^76^	http://www.bioconductor.org/packages/devel/bioc/html/ComplexHeatmap.html
**FGSEA R PACKAGE**	Subramanian et al.^77^	https://bioconductor.org/packages/release/bioc/html/fgsea.html
**BURROS WHEELER ALIGNER**	Li et al.^78^	http://maq.sourceforge.net/
**MODEL-BASED ANALYSIS OF CHIP-SEQ**	Zhang et al.^79^	http://liulab.dfci.harvard.edu/MACS/
**BEDTOOLS**	Quinlan et al.^80^	http://code.google.com/p/bedtools
**GREAT**	McLean^81^	http://great.stanford.edu/public/html/
**MARTIX-EQTL**	Shabalin^82^	https://CRAN.R-project.org/package=matrixEQTL

## Highlights

- Modulating sphingolipid metabolism affects the induction of trained immunity
- Inhibition of acid ceramidase effectively suppresses trained immunity
- Blocking acid ceramidase alters lipids, gene expression, and histone modifications
- SNPs around *ASAH1*, encoding acid ceramidase, associate with trained immunity
