# Supplementary material for: Acid ceramidase regulates innate immune memory
Source: Cell Rep. Author manuscript; Available in PMC 2025 Mar 14. (PMC11907240; doi:10.1016/j.celrep.2023.113458)
Supplement: supplemental 1 [file NIHMS1963467-supplement-supplemental_1.pdf]

Cell Reports, Volume *42*

Supplemental information

Acid ceramidase regulates innate immune memory

Nils Rother, Cansu Yanginlar, Geoffrey Prévot, Inge Jonkman, Maaike Jacobs, Mandy M.T. van Leent, Julia van Heck, Vasiliki Matzaraki, Anthony Azzun, Judit Morla-Folch, Anna Ranzenigo, William Wang, Roy van der Meel, Zahi A. Fayad, Niels P. Riksen, Luuk B. Hilbrands, Rik G.H. Lindeboom, Joost H.A. Martens, Michiel Vermeulen, Leo A.B. Joosten, Mihai G. Netea, Willem J.M. Mulder, Johan van der Vlag, Abraham J.P. Teunissen, and Raphaël Duivenvoorden

Supplementary Table 1: Sphingolipids used to formulate the sphingolipid-loaded nanobiologics. Related to Figure 1.

#	Sphingolipid name	Size (nm) of nanoparticle	Dispersity index of nanoparticle	Structure
1	Sphingosine-1-phosphate (d18:1/16:0)	29.8	0.239	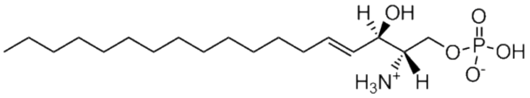
2	Sphingosine (d20:1)	45.3	0.196	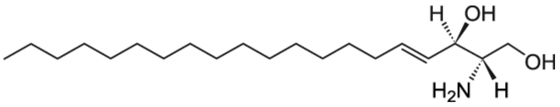
3	C24 Ceramide (d18:1/24:0)	59.5	0.208	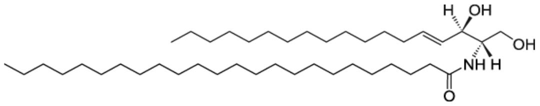
4	C16 Ceramide (d18:1/16:0)	32.7	0.242	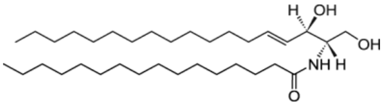
5	C16 Dihydroceramide (18:0/16:0)	59.7	0.165	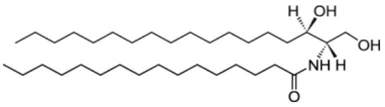
6	C24 Dihydroceramide (d18:0/24:0)	47.3	0.180	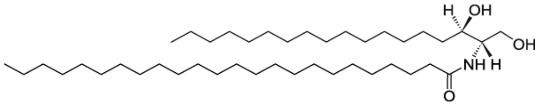
7	C16 Ceramide-1-phosphate (d18:1/16:0)	42.5	0.209	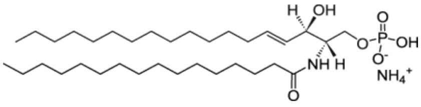
8	C18:1 Ceramide-1-phosphate (d18:1/18:1(9Z))	44.0	0.225	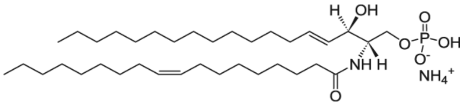
9	C12 Ceramide-1-phosphate (d18:1/12:0)	34.0	0.251	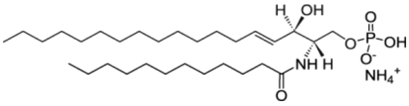
10	C16 Galactosyl(α) Ceramide (d18:1/16:0)	35.7	0.226	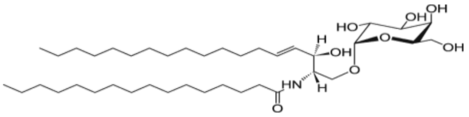

11	C16 Galactosyl(β) Ceramide (d18:1/16:0)	48.3	0.204	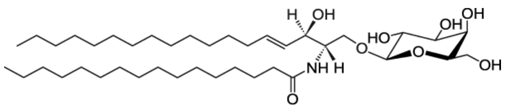
12	C24:0 Galactosyl(β) Ceramide (d18:1/24:0)	43.7	0.226	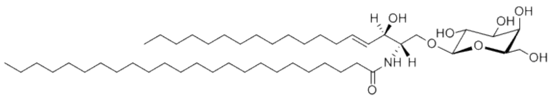
13	C24:1 Galactosyl(β) Ceramide (d18:1/24:1)	51.9	0.169	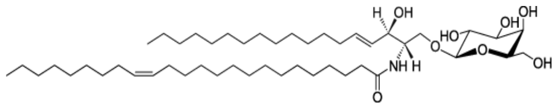
14	C24:0 Lactosyl(β) Ceramide (d18:1/24:0)	33.5	0.211	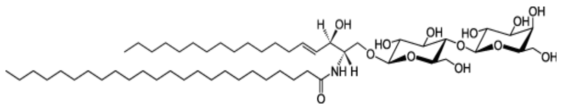
15	C24:1 Lactosyl(β) Ceramide (d18:1/24:1)	30.1	0.232	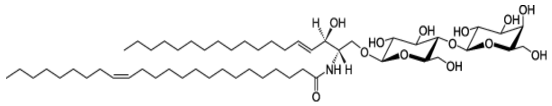
16	24:0 Sphingomyelin	96.3	0.105	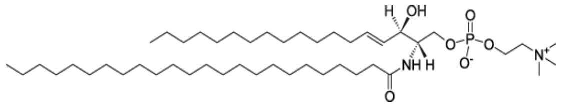
17	24:1 Sphingomyelin	40.6	0.217	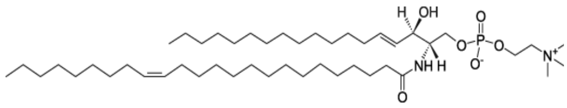
18	C18:0 Glucosyl(β) Ceramide (d18:1/18:0)	39.2	0.207	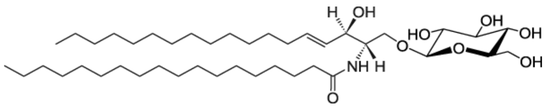
19	C18:1 Glucosyl(β) Ceramide (d18:1/18:1(9Z))	41.1	0.184	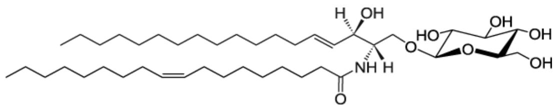
Ctrl	Unloaded nanobiologics	21.5	0.293	NA

Sizes are reported as the mean of the number average size distribution. Both nanoparticle size and dispersity index were determined by dynamic light scattering.

Supplementary Table 2: P-values for one-way ANOVA with Dunnett's post-test for data in Figures 1 D and E. Related to Figure 1.

Sphingolipid-NP	Versus RPMI		Versus empty NPs	
Figure 1D	TNF	IL-6	TNF	IL-6
#1	0.523	0.747	0.753	0.732
#2	0.998	0.092	0.999	0.091
#3	1.000	0.164	0.999	0.162
#4	1.000	5.72E-05	0.999	6.57E-05
#5	0.275	1.000	0.630	1.000
#6	1.000	1.000	1.000	1.000
#7	0.158	0.212	0.296	0.203
#8	0.986	1.000	0.999	1.000
#9	0.785	0.990	0.992	0.989
#10	1.000	1.000	0.923	1.000
#11	0.162	1.000	0.436	0.999
#12	0.312	1.000	0.683	1.000
#13	0.991	3.15E-04	0.781	2.92E-04
#14	0.800	1.000	0.993	0.999
#15	0.014	1.000	0.064	0.999
#16	0.646	0.046	0.959	0.045
#17	0.917	1.000	0.999	0.999
#18	1.000	0.402	1.000	0.399
#19	1.000	0.962	1.000	0.961
	Versus HKCA		Versus empty NPs	
Figure 1E				
#1	0.013	1.000	0.083	1.000
#2	0.025	0.292	0.718	0.936
#3	0.013	0.344	0.550	0.965
#4	0.242	1.58E-03	0.999	0.042
#5	2.62E-05	1.000	0.020	0.999
#6	0.068	1.000	0.937	0.806
#7	2.63E-03	0.018	0.026	0.019
#8	0.984	0.929	0.999	1.000
#9	1.15E-07	1.000	4.30E-04	0.896
#10	1.000	1.000	0.999	0.999
#11	8.63E-07	1.000	1.09E-03	0.999
#12	1.03E-06	0.999	1.58E-03	0.555
#13	1.000	4.98E-03	0.989	0.090
#14	1.000	0.017	0.996	0.207
#15	2.67E-11	0.849	8.73E-07	0.208
#16	8.50E-08	1.000	4.33E-04	0.999
#17	0.421	0.154	1.000	0.752
#18	1.40E-03	0.615	0.175	0.207
#19	1.000	1.34E-03	0.283	0.036

Figure S1

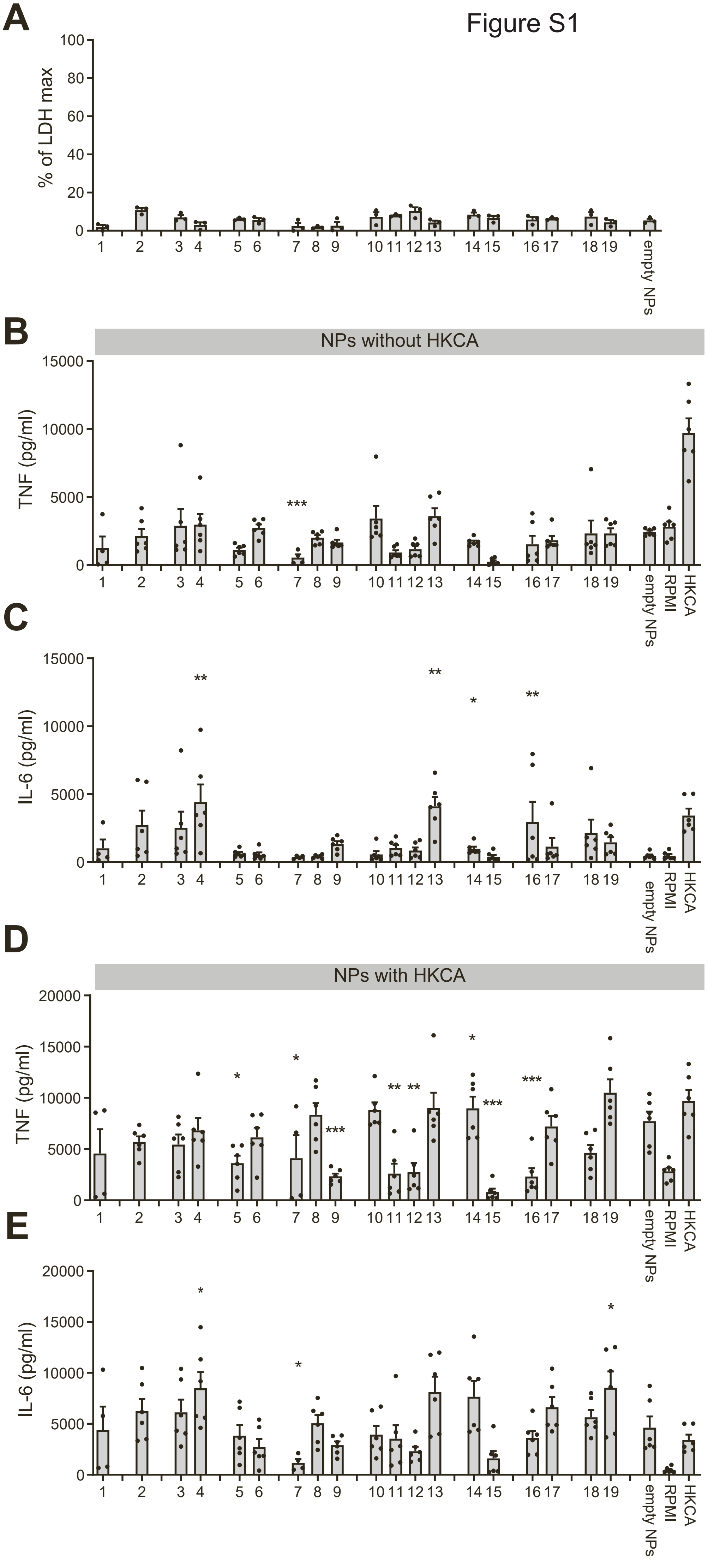
A
% of LDH max
100
80
60
40
20
0
1 2 3 4 5 6 7 8 9 10 11 12 13 14 15 16 17 18 19 empty NPs
B
NPs without HKCA
TNF (pg/ml)
15000
10000
5000
0
***
1 2 3 4 5 6 7 8 9 10 11 12 13 14 15 16 17 18 19 empty NPs RPMI HKCA
C
IL-6 (pg/ml)
15000
10000
5000
0
** ** * **
1 2 3 4 5 6 7 8 9 10 11 12 13 14 15 16 17 18 19 empty NPs RPMI HKCA
D
NPs with HKCA
TNF (pg/ml)
20000
15000
10000
5000
0
* * *** * ** ** *** ***
1 2 3 4 5 6 7 8 9 10 11 12 13 14 15 16 17 18 19 empty NPs RPMI HKCA
E
IL-6 (pg/ml)
20000
15000
10000
5000
0
* * *
1 2 3 4 5 6 7 8 9 10 11 12 13 14 15 16 17 18 19 empty NPs RPMI HKCA

Supplementary Figure 1: Sphingolipid-loaded nanobiologics toxicity and effect on innate immune memory. Related to Figure 1.

(A) Lactate dehydrogenase (LDH) measurement of PBMCs treated with sphingolipid-nanobiologics (50 µM) for 24 hours (n = 6 donors).

(B - E) PBMCs were stimulated for 24 hours with sphingolipid-nanobiologics alone (B, C) or in combination with HKCA (D, E). After a five-day resting period, cells were restimulated with LPS for 24 hours and cytokine production measured in the supernatant (n = 6 donors). Data are expressed as mean ± SEM. $^*p < 0.05$, $^{**}p < 0.01$, $^{***}p < 0.001$; p-values were calculated using one-way ANOVA with Dunnett's post-test

Figure S2

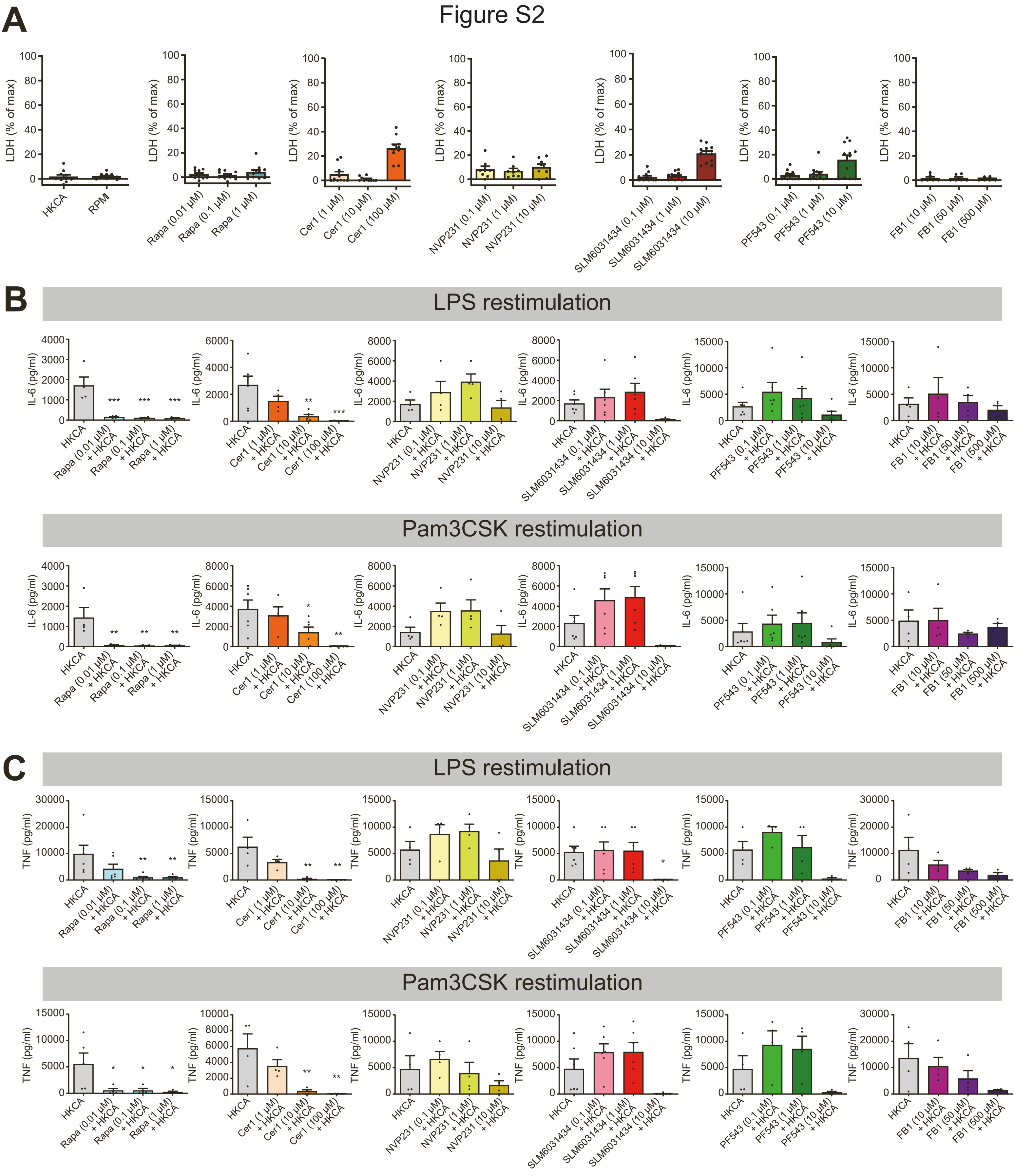
A
LDH (% of max)
HKCA
RPMI
Rapa (0.01 μM)
Rapa (0.1 μM)
Rapa (1 μM)
Cer1 (1 μM)
Cer1 (10 μM)
Cer1 (100 μM)
NVP231 (0.1 μM)
NVP231 (1 μM)
NVP231 (10 μM)
SLM6031434 (0.1 μM)
SLM6031434 (1 μM)
SLM6031434 (10 μM)
PF543 (0.1 μM)
PF543 (1 μM)
PF543 (10 μM)
FB1 (10 μM)
FB1 (50 μM)
FB1 (500 μM)
B
LPS restimulation
Pam3CSK restimulation
IL-6 (pg/ml)
+ HKCA
C
LPS restimulation
Pam3CSK restimulation
TNF (pg/ml)

Supplementary Figure 2: Dose finding experiments of sphingolipid inhibitors. Related to Figure 2.

(A) Lactate dehydrogenase (LDH) release after 24 hour long stimulation with sphingolipid enzyme inhibitors (n = 10 donors for ceranib-1 (Cer1), n = 6 donors for fumonisin B1 (FB1), n = 8 donors for NVP231, n = 12 donors for PF543 and SLM6031434, n = 11 donors for rapamycin (Rapa)).

(B, C) Dose finding training experiments with sphingolipid inhibitors. PBMCs were stimulated with HKCA and different doses of specified inhibtors, or RPMI as control. After a five-day resting period, cells were restimulated with LPS or Pam3CSK and cytokine production of IL-6 (B) and TNF (C) was measured in the supernatant by ELISA (n = 4 donors for rapamycin (Rapa), NVP231 and fumonisin B1 (FB1), n = 6 donors for ceranib-1, SLM6031434 and PF543). Data are expressed as mean ± SEM. *p < 0.05, **p < 0.01, ***p < 0.001; p-values were calculated using one-way ANOVA with Dunnett's post-test.

Figure S3

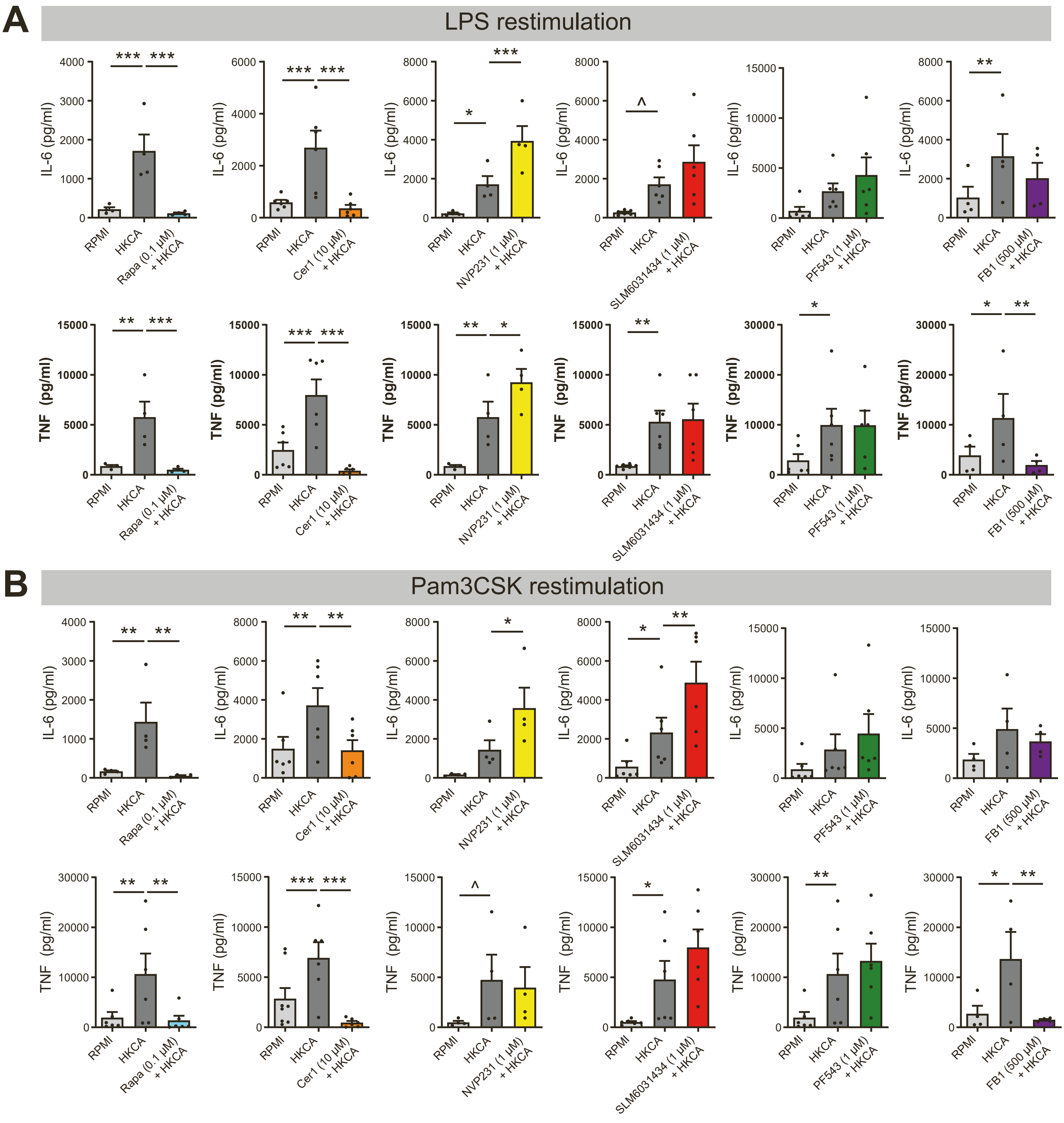
A
LPS restimulation
IL-6 (pg/ml)
TNF (pg/ml)
RPMI
HKCA
Rapa (0.1 µM) + HKCA
Cer1 (10 µM) + HKCA
NVP231 (1 µM) + HKCA
SLM6031434 (1 µM) + HKCA
PF543 (1 µM) + HKCA
FB1 (500 µM) + HKCA
B
Pam3CSK restimulation

Supplementary Figure 3: Sphingolipid enzyme inhibitor effect on innate immune memory. Related to Figure 2.

(A, B) PBMCs were stimulated for 24 hours with HKCA alone, with HKCA together with specified inhibitors, or RPMI as control. After a five-day resting period, cells were restimulated for 24 hours with LPS (A) or Pam3CSK (B) and cytokine production measured in the supernatant by ELISA (n = 4 donors for fumonisin B1 (FB1) and NVP231, n = 6 donors for ceranib-1, rapamycin (Rapa), PF543 and SLM6031434).

^p < 0.06, *p<0.05, **p<0.01, ***p<0.001 for one-way ANOVA with Dunnett's post-test. Data are represented as mean ± SEM.

Figure S4

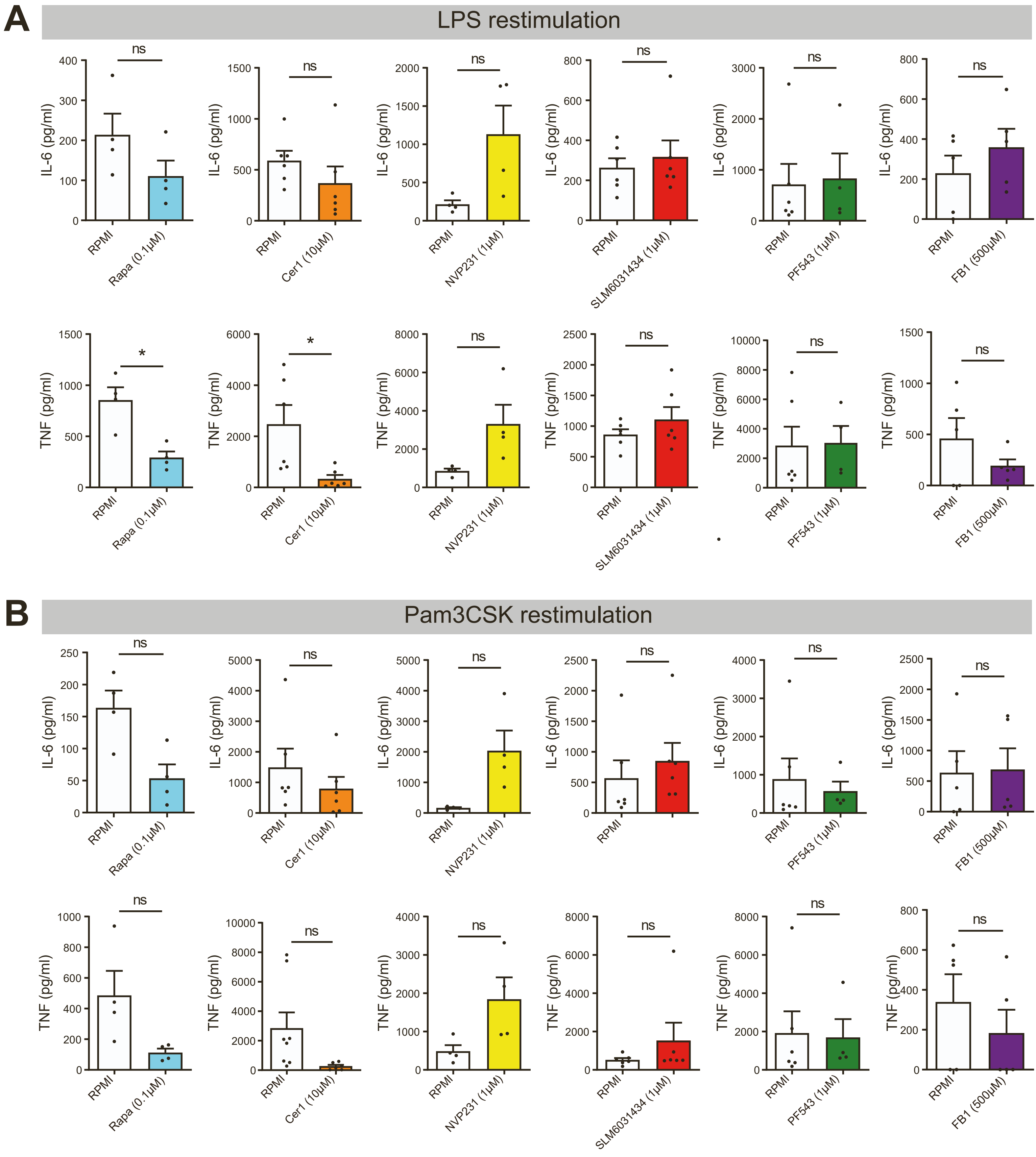
A
LPS restimulation
IL-6 (pg/ml)
TNF (pg/ml)
RPMI
Rapa (0.1µM)
Cer1 (10µM)
NVP231 (1µM)
SLM6031434 (1µM)
PF543 (1µM)
FB1 (500µM)
ns
*
B
Pam3CSK restimulation
IL-6 (pg/ml)
TNF (pg/ml)
RPMI
Rapa (0.1µM)
Cer1 (10µM)
NVP231 (1µM)
SLM6031434 (1µM)
PF543 (1µM)
FB1 (500µM)
ns

Supplementary Figure 4: Sphingolipid enzyme inhibitor effect on cytokine production. Related to Figure 2.

(A, B) PBMCs were stimulated for 24 hours with specified inhibitors, or RPMI as control. After a five-day resting period, cells were restimulated for 24 hours with LPS (A) or Pam3CSK (B) and cytokine production measured in the supernatant by ELISA (n = 6 donors for ceranib-1, PF543 and SLM6031434, n = 4 donors for NVP231 and rapamycin (Rapa), n = 5 donors for fumonisin B1 (FB1)). *$p<0.05$ for two-tailed paired t-test. Data are represented as mean ± SEM.

Figure S5

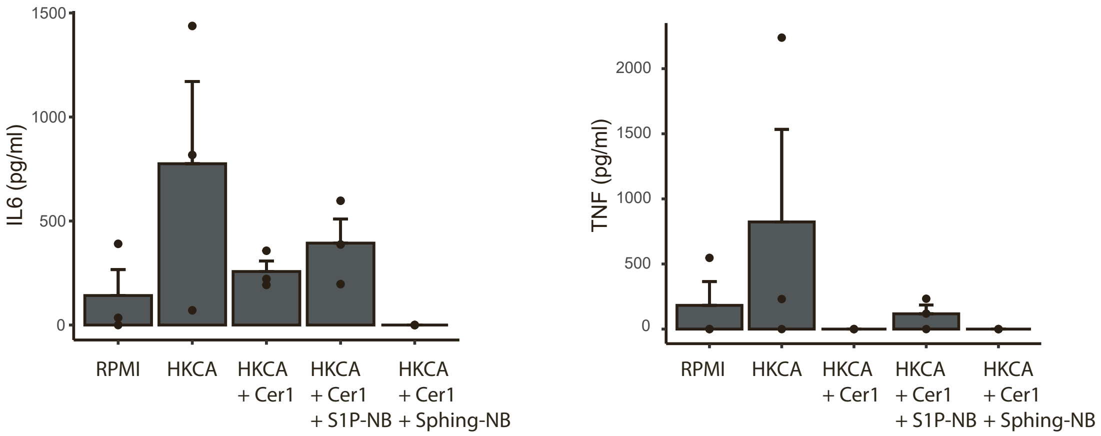
IL6 (pg/ml)
1500
1000
500
0
RPMI
HKCA
HKCA
+ Cer1
HKCA
+ Cer1
+ S1P-NB
HKCA
+ Cer1
+ Sphing-NB
TNF (pg/ml)
2000
1500
1000
500
0
RPMI
HKCA
HKCA
+ Cer1
HKCA
+ Cer1
+ S1P-NB
HKCA
+ Cer1
+ Sphing-NB

Supplementary Figure 5: Addition of sphingosine or sphingosine-1-phosphate cannot rescue training upon acid ceramidase inhibition. Related to Figure 2.

PBMCs were stimulated for 24 hours with HKCA alone, with HKCA together with ceranib-1 and in combination of HKCA, ceranib-1 alone and sphingosine or sphingosine-1-phosphate containing nanobiologics or RPMI as control. After a five-day resting period, cells were restimulated for 24 hours with LPS and cytokine production measured in the supernatant by ELISA (n = 3 donors). Data are represented as mean ± SEM.

Figure S6

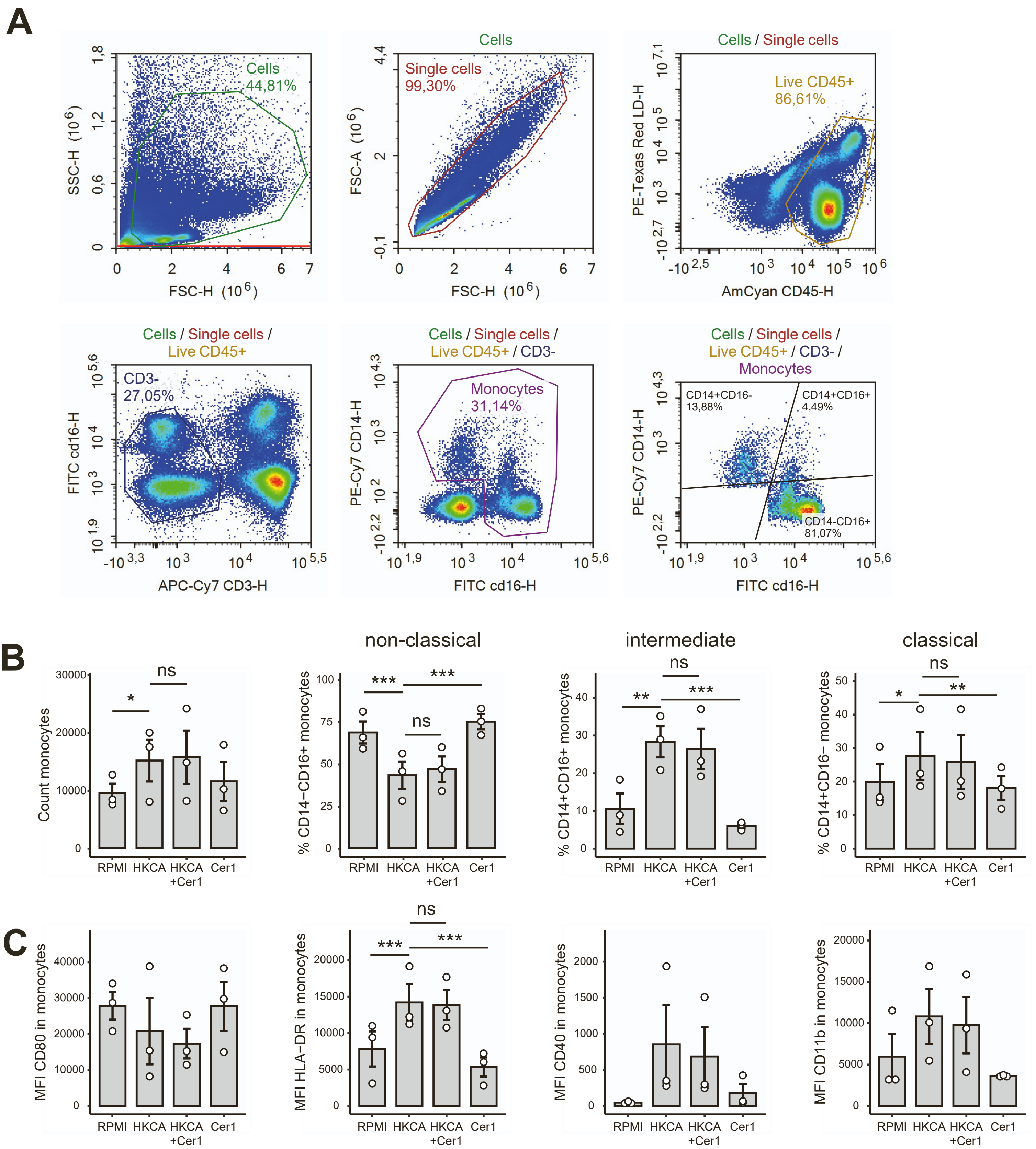

A
Cells
44,81%
SSC-H (10^6)
FSC-H (10^6)
Cells
Single cells
99,30%
FSC-A (10^6)
FSC-H (10^6)
Cells / Single cells
Live CD45+
86,61%
PE-Texas Red LD-H
AmCyan CD45-H
Cells / Single cells / Live CD45+
CD3-
27,05%
FITC cd16-H
APC-Cy7 CD3-H
Cells / Single cells / Live CD45+ / CD3-
Monocytes
31,14%
PE-Cy7 CD14-H
FITC cd16-H
Cells / Single cells / Live CD45+ / CD3- / Monocytes
CD14+CD16-
13,88%
CD14+CD16+
4,49%
CD14-CD16+
81,07%
PE-Cy7 CD14-H
FITC cd16-H
B
non-classical
intermediate
classical
Count monocytes
% CD14−CD16+ monocytes
% CD14+CD16+ monocytes
% CD14+CD16− monocytes
ns
*
***
**
RPMI
HKCA
HKCA +Cer1
Cer1
C
MFI CD80 in monocytes
MFI HLA-DR in monocytes
MFI CD40 in monocytes
MFI CD11b in monocytes
RPMI
HKCA
HKCA +Cer1
Cer1

Supplementary Figure 6: Monocyte differentiation is not affected by inhibition of acid ceramidase. Related to Figure 2.

(A) Gating strategy of monocytes and monocyte subsets.

(B and C) PBMCs were stimulated for 24 hours with HKCA alone, with HKCA together with ceranib-1, ceranib-1 alone or RPMI as control. After a five-day resting period flow cytometric analysis was performed. Absolute monocyte count as detected during flow cytometric acquisition, percentage of monocyte subsets and mean fluorescence intensity of surface molecules are depicted as mean ± SEM (n = 3 donors).

*p<0.05, *p<0.01, ***p<0.001 for one-way ANOVA with Dunnett's post-test compared to HKCA.

Figure S7

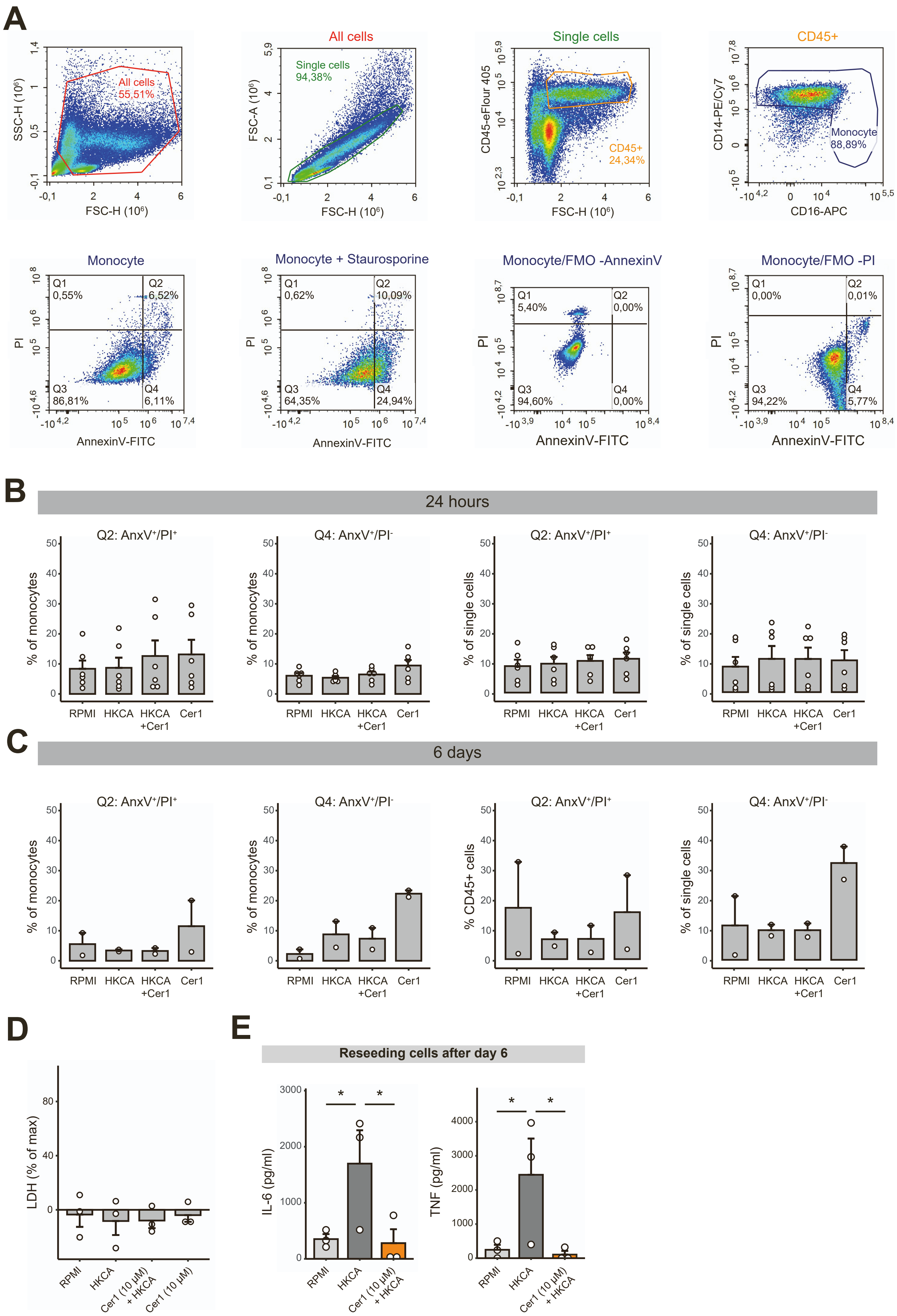
A
All cells
55,51%
Single cells
94,38%
CD45+
24,34%
Monocyte
88,89%
SSC-H (10⁶)
FSC-H (10⁶)
FSC-A (10⁶)
CD45-eFlour 405
CD14-PE/Cy7
CD16-APC
Monocyte
Monocyte + Staurosporine
Monocyte/FMO -AnnexinV
Monocyte/FMO -PI
PI
AnnexinV-FITC
Q1 0,55%
Q2 6,52%
Q3 86,81%
Q4 6,11%
Q1 0,62%
Q2 10,09%
Q3 64,35%
Q4 24,94%
Q1 5,40%
Q2 0,00%
Q3 94,60%
Q4 0,00%
Q1 0,00%
Q2 0,01%
Q3 94,22%
Q4 5,77%
B
24 hours
Q2: AnxV+/PI+
Q4: AnxV+/PI-
% of monocytes
% of single cells
RPMI
HKCA
HKCA +Cer1
Cer1
C
6 days
% CD45+ cells
D
LDH (% of max)
Cer1 (10 µM) + HKCA
Cer1 (10 µM)
E
Reseeding cells after day 6
IL-6 (pg/ml)
TNF (pg/ml)

Supplementary Figure 7: No induction of cell death by inhibition of acid ceramidase. Related to Figure 2.

(A) Gating strategy of apoptotic (AnxV+/PI-) and necrotic (AnxV+/PI+) cells. Gating for AnnexinV and PI was determined based on staurosporine treated samples (apoptosis inducing positive control) and FMO's (fluorophore minus one) for AnnexinV and PI.

(B, C) PBMCs were analyzed after 24 hour stimulation with HKCA alone, with HKCA together with ceranib-1, ceranib-1 alone or RPMI as control (B) or after a 5-day resting period following initial stimulation (C). Flow cytometric analysis was performed to identify apoptotic and necrotic cells in monocytes (CD16+/CD14+) and CD45+ cells (n = 6 donors for 24 h timepoint and n = 3 donors for 6 day timepoint).

Data were analyzed with a one-way ANOVA with Dunnett's post-test compared to HKCA. Data are presented as mean ± SEM.

(D) LDH analysis of samples after the resting period.

(E) Cells were collected after the resting period, counted and replated in equal numbers (50,000 cells per condition) before restimulation with LPS. Cytokine response was measured using ELISA (n = 3 donors). *$p<0.05$ for one-way ANOVA with Dunnett's post-test compared to HKCA. Data are represented as mean + SEM.

Figure S8

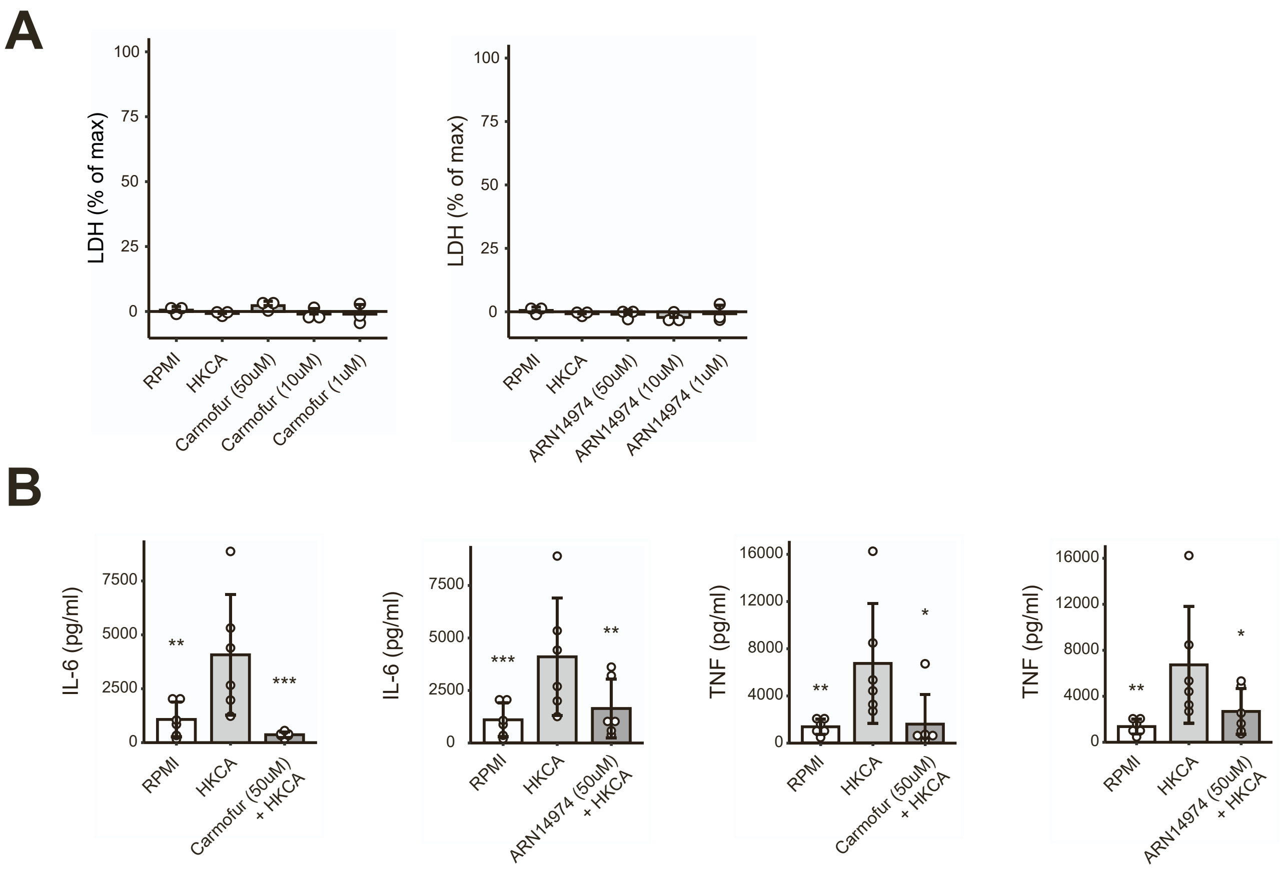
A
100
75
50
25
0
LDH (% of max)
RPMI
HKCA
Carmofur (50uM)
Carmofur (10uM)
Carmofur (1uM)
ARN14974 (50uM)
ARN14974 (10uM)
ARN14974 (1uM)
B
IL-6 (pg/ml)
7500
5000
2500
**
***
Carmofur (50uM) + HKCA
ARN14974 (50uM) + HKCA
TNF (pg/ml)
16000
12000
8000
4000
*

Supplementary Figure 8: Effect of alternative acid ceramidase inhibitors on trained immunity. Related to Figure 2.

(A) Lactate dehydrogenase (LDH) release after 24 hour long stimulation with sphingolipid enzyme inhibitors (n = 3 donors).

(B) PBMCs were stimulated for 24 hours with HKCA alone, with HKCA together with specified inhibitors, or RPMI as control. After a five-day resting period, cells were restimulated for 24 hours with LPS and cytokine production measured in the supernatant by ELISA (n = 6 donors).

**p<0.01, ***p<0.001 for one-way ANOVA with Dunnett's post-test compared to HKCA. Data are represented as mean ± SEM.

Figure S9

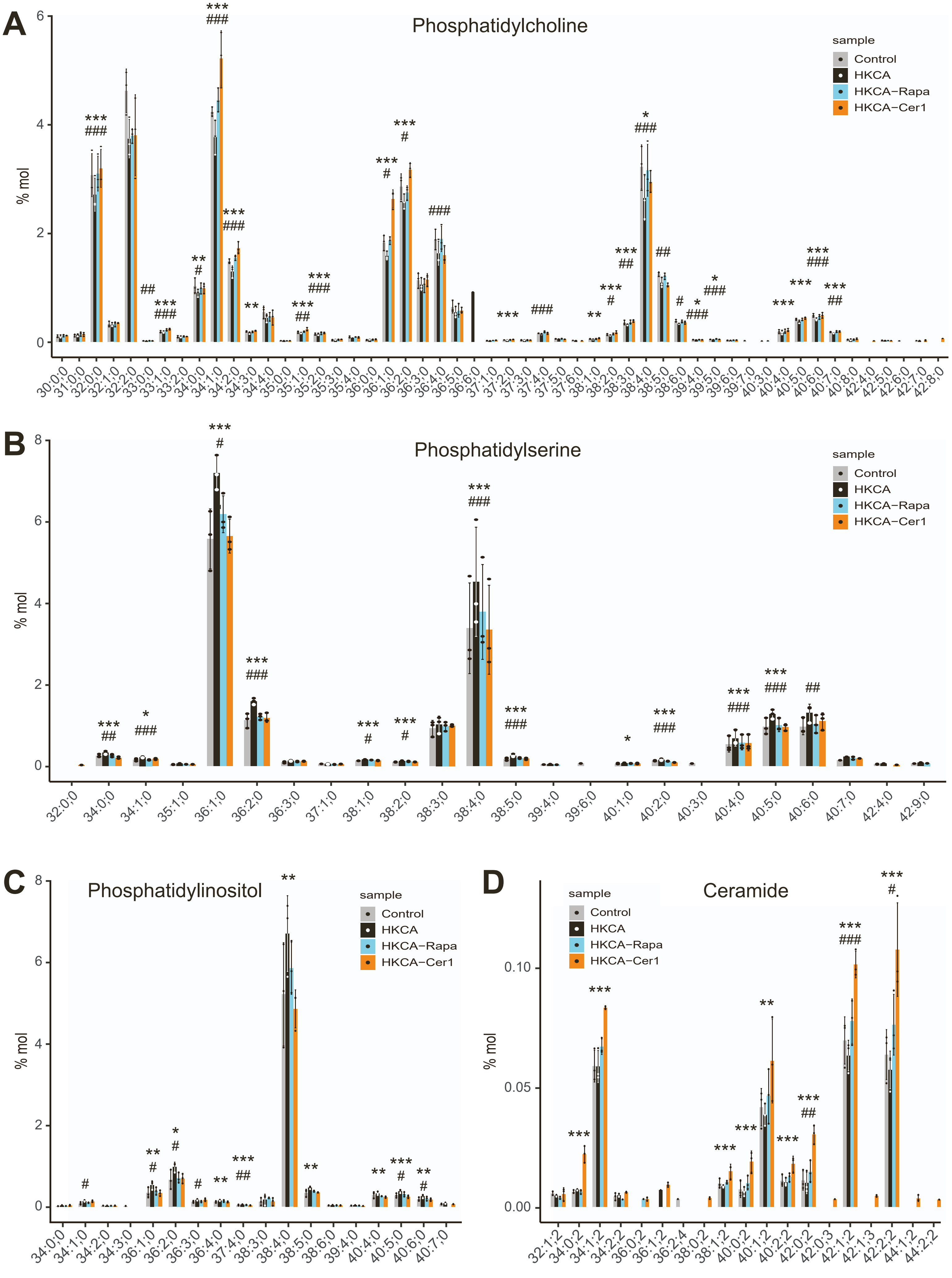
A
Phosphatidylcholine
% mol
sample
Control
HKCA
HKCA−Rapa
HKCA−Cer1
30:0;0 31:0;0 32:0;0 32:1;0 32:2;0 33:0;0 33:1;0 33:2;0 34:0;0 34:1;0 34:2;0 34:3;0 34:4;0 35:0;0 35:1;0 35:2;0 35:3;0 35:4;0 36:0;0 36:1;0 36:2;0 36:3;0 36:4;0 36:5;0 36:6;0 37:1;0 37:2;0 37:3;0 37:4;0 37:5;0 37:6;0 38:1;0 38:2;0 38:3;0 38:4;0 38:5;0 38:6;0 39:4;0 39:5;0 39:6;0 39:7;0 40:3;0 40:4;0 40:5;0 40:6;0 40:7;0 40:8;0 42:4;0 42:5;0 42:6;0 42:7;0 42:8;0
B
Phosphatidylserine
% mol
sample
Control
HKCA
HKCA−Rapa
HKCA−Cer1
32:0;0 34:0;0 34:1;0 35:1;0 36:1;0 36:2;0 36:3;0 37:1;0 38:1;0 38:2;0 38:3;0 38:4;0 38:5;0 39:4;0 39:6;0 40:1;0 40:2;0 40:3;0 40:4;0 40:5;0 40:6;0 40:7;0 42:4;0 42:9;0
C
Phosphatidylinositol
% mol
sample
Control
HKCA
HKCA−Rapa
HKCA−Cer1
34:0;0 34:1;0 34:2;0 34:3;0 36:1;0 36:2;0 36:3;0 36:4;0 37:4;0 38:3;0 38:4;0 38:5;0 38:6;0 39:4;0 40:4;0 40:5;0 40:6;0 40:7;0
D
Ceramide
% mol
sample
Control
HKCA
HKCA−Rapa
HKCA−Cer1
32:1;2 34:0;2 34:1;2 34:2;2 36:0;2 36:1;2 36:2;4 38:0;2 38:1;2 40:0;2 40:1;2 40:2;2 42:0;2 42:0;3 42:1;2 42:1;3 42:2;2 44:1;2 44:2;2

Supplementary Figure 9: Changes in lipid subspecies in response to acid ceramidase inhibition. Related to Figure 4.

(A - D) PBMCs were stimulated for 24 hours with HKCA, HKCA + rapamycin (Rapa), HKCA + ceranib-1 (Cer1), or RPMI as control. The monocytes were subsequently purified and analyzed for their lipid content by mass spectrometry-based shotgun lipidomics. (n = 3 donors per treatment group). Abundance of phosphatidylcholine (A), phosphatidylserine (B), phosphatidylinositol (C) and ceramide (D) lipid subspecies as molar percentage of all lipids per treatment group. Data are represented as mean ± SD. *p < 0.05, **p < 0.01, ***p < 0.001 between HKCA and HKCA + ceranib-1; $^{\#}p<0.05$, $^{\#\#}p<0.01$, $^{\#\#\#}p < 0.001$ between HKCA and HKCA + rapamycin as determined by one-way ANOVA with Dunnetts post-test.

Figure S10

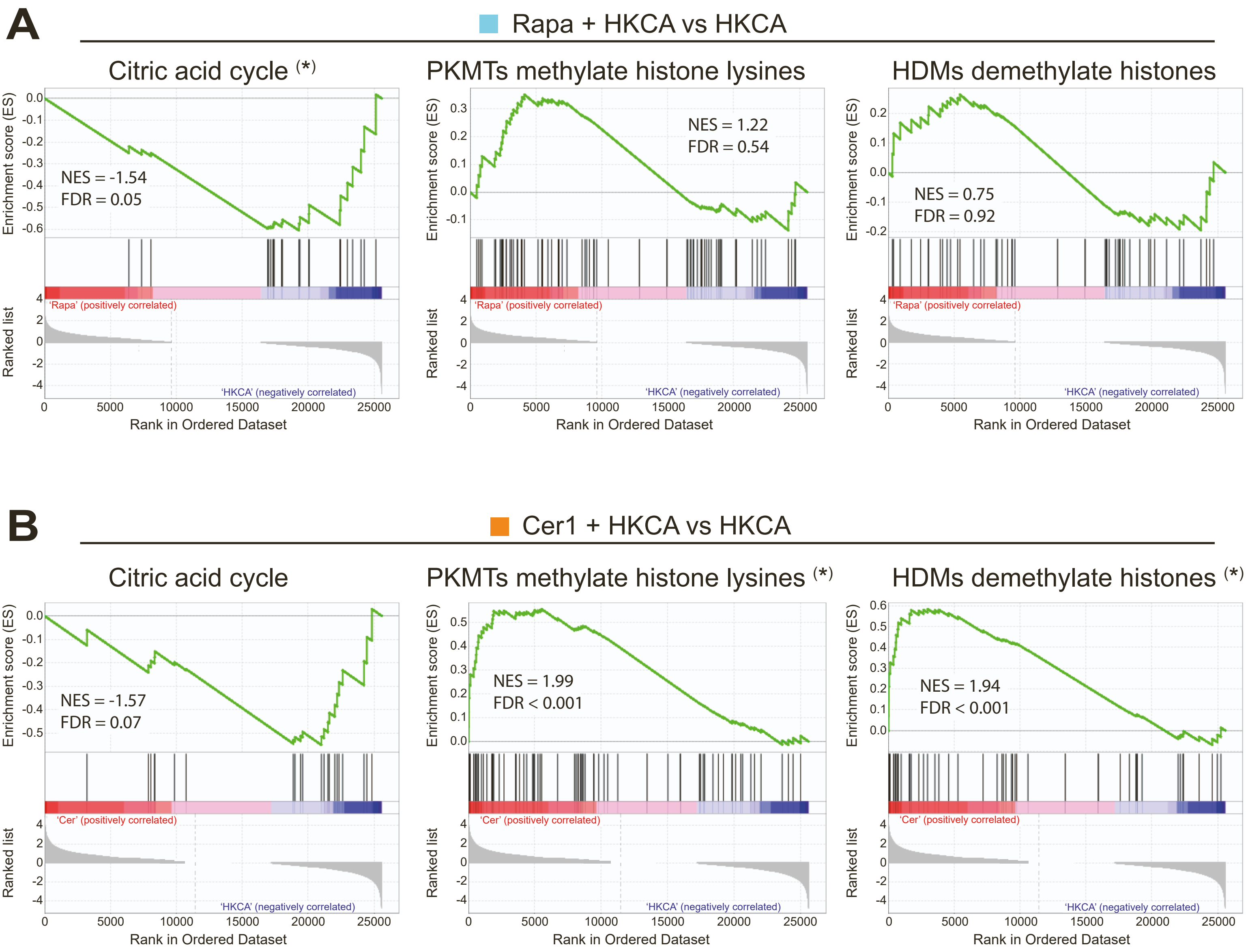
A
Rapa + HKCA vs HKCA
Citric acid cycle (*)
NES = -1.54
FDR = 0.05
PKMTs methylate histone lysines
NES = 1.22
FDR = 0.54
HDMs demethylate histones
NES = 0.75
FDR = 0.92
Enrichment score (ES)
Ranked list
'Rapa' (positively correlated)
'HKCA' (negatively correlated)
Rank in Ordered Dataset
B
Cer1 + HKCA vs HKCA
Citric acid cycle
NES = -1.57
FDR = 0.07
PKMTs methylate histone lysines (*)
NES = 1.99
FDR < 0.001
HDMs demethylate histones (*)
NES = 1.94
FDR < 0.001
Enrichment score (ES)
Ranked list
'Cer' (positively correlated)
'HKCA' (negatively correlated)
Rank in Ordered Dataset

Supplementary Figure 10: The effect of acid ceramidase inhibition on the monocytes' transcriptome. Related to Figure 5.

(A, B) PBMCs were stimulated for 24 hours with RPMI, HKCA, HKCA + rapamycin (Rapa) or HKCA + ceranib-1 (Cer1), after which cells were rested for 5 days. Subsequently, monocytes were purified and transcriptome analysis performed. (n = 3 donors per treatment). Gene set enrichment analysis for reactome gene sets of 'Citric acid cycle' (left panels), 'PKMTs methylate histone lysines' (middle panels) and 'HDMs demethylate histones' (right panels) is shown. Asterisks indicate gene sets with significant FDR. Abbreviations: NES: Normalized enrichment score, FDR: False discovery rate, PKMTs: Protein lysine methyltransferases, HDM: histone demethylases.

Figure S11

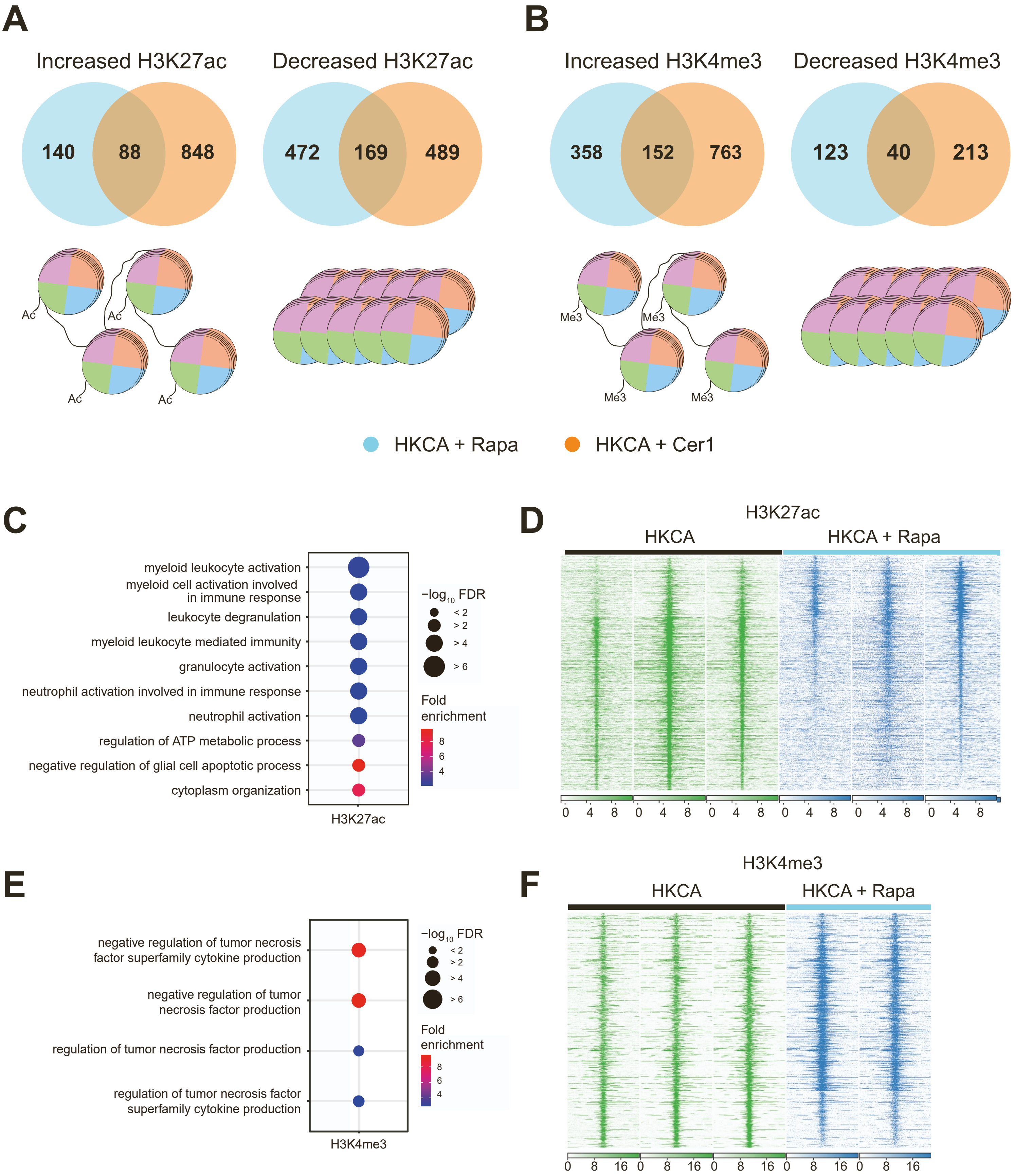

A
Increased H3K27ac
140
88
848
Decreased H3K27ac
472
169
489
Ac
B
Increased H3K4me3
358
152
763
Decreased H3K4me3
123
40
213
Me3
HKCA + Rapa
HKCA + Cer1
C
myeloid leukocyte activation
myeloid cell activation involved in immune response
leukocyte degranulation
myeloid leukocyte mediated immunity
granulocyte activation
neutrophil activation involved in immune response
neutrophil activation
regulation of ATP metabolic process
negative regulation of glial cell apoptotic process
cytoplasm organization
H3K27ac
−log10 FDR
< 2
> 2
> 4
> 6
Fold enrichment
8
6
4
D
H3K27ac
HKCA
HKCA + Rapa
0 4 8 0 4 8 0 4 8 0 4 8 0 4 8 0 4 8
E
negative regulation of tumor necrosis factor superfamily cytokine production
negative regulation of tumor necrosis factor production
regulation of tumor necrosis factor production
regulation of tumor necrosis factor superfamily cytokine production
H3K4me3
−log10 FDR
< 2
> 2
> 4
> 6
Fold enrichment
8
6
4
F
H3K4me3
HKCA
HKCA + Rapa
0 8 16 0 8 16 0 8 16 0 8 16 0 8 16

Supplementary Figure 11: Epigenetic changes in response to acid ceramidase inhibition. Related to Figure 6.

(A - F) PBMCs were stimulated for 24 hours with RPMI, HKCA, HKCA + rapamycin (Rapa) or HKCA + ceranib-1 (Cer1), after which the cells were rested for five days. The monocytes were subsequently purified and chromatin-immunoprecipitation performed for H3K27ac and H3K4me3 (n = 3 donors per treatment group).

(A, B) Venn Diagrams showing overlap of up or down regulated H3K27ac (A) and H3K4me3 markers (B) in HKCA + rapamycin (Rapa) and HKCA + ceranib-1 (Cer1) treated PMBCs compared to those treated with HKCA alone (Fold change > 2,5, p < 0,1).

(C) GO Biological processes associated with genes located in proximity to dynamic H3K27ac ChIP-seq peaks in HKCA + rapamycin treated cells compared to HKCA-trained cells (fold change > 2.5, FDR < 0.1).

(D) Heatmaps showing the H3K27ac peak signals (RPKM) in a 10 kilobase window around significant dynamic regions (fold change > 2.5, p< 0.1).

(E) GO Biological processes associated with genes located in proximity to dynamic H3K4me3 ChIP-seq peaks in HKCA + rapamycin treated cells compared to HKCA-trained cells (Fold change > 2).

(F) Heatmaps showing the H3K4me3 peak signals (RPKM) in a 10 kilobase window around significant dynamic regions (Fold change > 2).

RPKM: Reads per kilobase per million mapped reads.

Figure S12

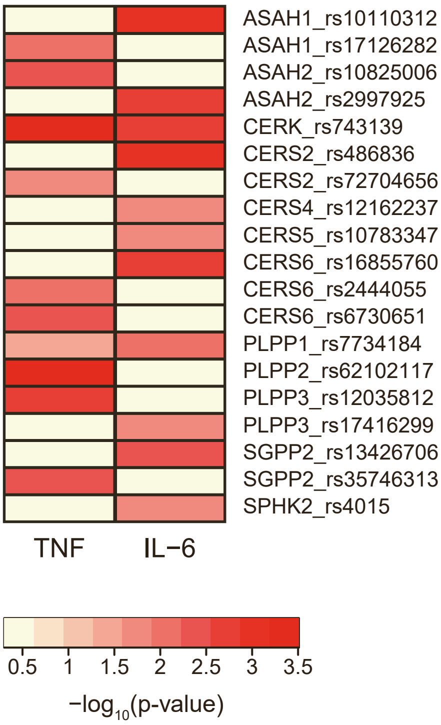
ASAH1_rs10110312
ASAH1_rs17126282
ASAH2_rs10825006
ASAH2_rs2997925
CERK_rs743139
CERS2_rs486836
CERS2_rs72704656
CERS4_rs12162237
CERS5_rs10783347
CERS6_rs16855760
CERS6_rs2444055
CERS6_rs6730651
PLPP1_rs7734184
PLPP2_rs62102117
PLPP3_rs12035812
PLPP3_rs17416299
SGPP2_rs13426706
SGPP2_rs35746313
SPHK2_rs4015
TNF
IL−6
0.5
1
1.5
2
2.5
3
3.5
−log10(p-value)

Supplementary Figure 12: Single nucleotide polymorphisms (SNPs) around genes involved in sphingolipid metabolism associate with trained immunity cytokine responses. Related to Figure 7.

P-values of association between SNPs mapped to genes involved in the sphingolipid metabolism and the magnitude of cytokine production of *in vitro* trained monocytes (n = 251 healthy individuals for IL-6, n = 238 healthy individuals for TNF).
